# Loss of Mitogen-Activated Protein Kinase Kinase Kinase 4 (MAP3K4) Reveals a Requirement for MAPK Signalling in Mouse Sex Determination

**DOI:** 10.1371/journal.pbio.1000196

**Published:** 2009-09-15

**Authors:** Debora Bogani, Pam Siggers, Rachel Brixey, Nick Warr, Sarah Beddow, Jessica Edwards, Debbie Williams, Dagmar Wilhelm, Peter Koopman, Richard A. Flavell, Hongbo Chi, Harry Ostrer, Sara Wells, Michael Cheeseman, Andy Greenfield

**Affiliations:** 1 Mammalian Genetics Unit, Medical Research Council (MRC) Harwell, Oxfordshire, United Kingdom; 2 The Institute of Molecular Bioscience, University of Queensland, Brisbane, Australia; 3 Department of Immunobiology, Yale University School of Medicine, New Haven, Connecticut, United States of America; 4 Department of Immunology, St. Jude Children's Research Hospital, Memphis, Tennessee, United States of America; 5 Human Genetics Program, New York University School of Medicine, New York, New York, United States of America; 6 The Mary Lyon Centre, Medical Research Council (MRC) Harwell, Oxfordshire, United Kingdom; National Institute for Medical Research, United Kingdom

## Abstract

Sex determination in mammals is controlled by the presence or absence of the Y-linked gene *SRY*. In the developing male (XY) gonad, sex-determining region of the Y (SRY) protein acts to up-regulate expression of the related gene, *SOX9*, a transcriptional regulator that in turn initiates a downstream pathway of testis development, whilst also suppressing ovary development. Despite the requirement for a number of transcription factors and secreted signalling molecules in sex determination, intracellular signalling components functioning in this process have not been defined. Here we report a role for the phylogenetically ancient mitogen-activated protein kinase (MAPK) signalling pathway in mouse sex determination. Using a forward genetic screen, we identified the recessive boygirl (*byg*) mutation. On the C57BL/6J background, embryos homozygous for *byg* exhibit consistent XY gonadal sex reversal. The *byg* mutation is an A to T transversion causing a premature stop codon in the gene encoding MAP3K4 (also known as MEKK4), a mitogen-activated protein kinase kinase kinase. Analysis of XY *byg/byg* gonads at 11.5 d post coitum reveals a growth deficit and a failure to support mesonephric cell migration, both early cellular processes normally associated with testis development. Expression analysis of mutant XY gonads at the same stage also reveals a dramatic reduction in *Sox9* and, crucially, *Sry* at the transcript and protein levels. Moreover, we describe experiments showing the presence of activated MKK4, a direct target of MAP3K4, and activated p38 in the coelomic region of the XY gonad at 11.5 d post coitum, establishing a link between MAPK signalling in proliferating gonadal somatic cells and regulation of *Sry* expression. Finally, we provide evidence that haploinsufficiency for *Map3k4* accounts for T-associated sex reversal (*Tas*). These data demonstrate that MAP3K4-dependent signalling events are required for normal expression of *Sry* during testis development, and create a novel entry point into the molecular and cellular mechanisms underlying sex determination in mice and disorders of sexual development in humans.

## Abstract 2

The *boygirl* (*byg*) mouse mutant reveals that MAP3K4-mediated signaling is necessary for normal *SRY* expression and testis specification in the developing mouse gonad.

## Author Summary

In mammals, whether an individual develops as a male or female depends on its sex chromosome constitution: those with a Y chromosome become males because of the development of the embryonic gonad into a testis. The Y-linked sex determining gene *SRY* regulates this process by initiating a pathway of gene and protein expression, including the expression of critical autosomal genes such as *SOX9*. We identified a mouse mutant that causes embryonic gonadal sex reversal: the development of ovaries in an XY embryo. This mutant, which we called boygirl (*byg*), was shown to contain an early stop codon that disrupts the autosomal gene encoding MAP3K4, a component of the mitogen-activated protein kinase (MAPK) signaling pathway. Analysis of embryonic XY gonads suggests that sex reversal is caused by delayed and reduced expression of the sex-determining gene *SRY*. Our data indicate, for the first time, a requirement for MAPK signaling in the developing XY gonad in order to facilitate normal expression of *SRY* and the downstream testis-determining genes and also suggest that reduced dosage of MAP3K4 may be the cause of a previously described autosomal sex-reversing mutation in the mouse. We predict that loss of MAP3K4 or other MAPK components may underlie disorders of sexual development (DSD) in humans as well.

## Introduction

Sex determination is the process by which an embryo develops into a male or female, namely, the formation of testes in an XY embryo and ovaries in an XX embryo. In the mouse, this process begins with commitment of cells of the bipotential genital ridge to either the testicular or ovarian fate at 11.5 d post coitum (dpc) [1]. In mammals such as mice and humans, this commitment depends on the presence or absence of the Y-linked testis-determining gene, *SRY* [2]–[4].

During the search for the elusive mammalian testis-determining factor, it was a criterion of correct identification that any candidate gene be associated with mutations that cause pure (gonadal) XY sex reversal: the development of an ovary in an XY individual. Such mutations in *SRY* were readily discovered in mice [5] and humans [6] exhibiting sex reversal, and this link with sex reversal has been a constant theme in the subsequent identification of novel, mostly autosomal, genes functioning in sex determination.

Instances of XY sex reversal in the mouse associated with single gene mutations remain relatively uncommon. Excluding *Sry*, they include targeted mutations of *Sox9* [7],[8], *Dax1* [9], *Fgf9* [10], *Fgfr2* [11],[12], *Gata4/Fog2* [13],[14], *Cbx2 (M33)* [15], and *Wt1(+KTS)* [16]. Mice harbouring targeted mutations in three members of the insulin-receptor signalling pathway also exhibit XY sex reversal [17]. In several of these cases, variability exists in the degree of sex reversal observed, depending on genomic context. The C57BL/6J background often biases gonadal development in favour of ovarian tissue in mutant XY embryos and this “B6 sensitivity” increases still further if the AKR/J Y chromosome (Y^AKR^) is present [14]. Additional genes have been identified that disrupt testis development, affecting testis cord formation or the differentiation of testis-specific cell lineages. These include *Dhh* [18]–[20], *Pdgfra* [21], *Pod1* [22], *Arx* [23], *Wnt4* [24], and *Spry2* [25]. The contribution of other protestis genes to sex determination, such as *Sf1* [26], *Dmrt1* [27], and *Sox8* [7], can be difficult to discern owing to functions of such genes earlier in gonad development or functional redundancy.

In addition to the contribution of specific genes, other autosomal loci have been reported to control sex determination in the mouse. Such loci have been identified on the basis of genetic segregation in cases of sex reversal observed when the Y chromosome of the C57BL/6 strain is replaced by that of *Mus domesticus poschiavinus* [28], or on the basis of their modifying the phenotypic effect of another sex determining locus [29],[30].

The search for novel sex determining genes has been driven in recent years by the transcriptional properties of candidate genes identified by expression profiling [31]–[35]. However, such gene-driven approaches have not yielded a significant number of novel sex reversal phenotypes or abnormalities of gonadal differentiation that could act as important models for the investigation of the molecular genetic basis of sex determination. Notable exceptions to this general observation include the genes *Cyp26b1* [36],[37] and *Pgds* [38],[39], whose roles in germ cell and somatic cell development, respectively, were established partly on the basis of earlier observations on their male-specific expression derived from systematic expression screens.

As an alternative to expression-based screens, we have employed a forward genetic approach to identifying loci controlling sexual development in the mouse. Using *N*-ethyl-*N*-nitrosourea (ENU) mutagenesis and a three-generation (G3) breeding scheme, we screened for abnormalities of the developing gonads in embryos homozygous for induced mutations. In one mutant pedigree, RECB/31, we identified XY embryos exhibiting abnormal testis cord development and, in some cases, gonadal sex reversal. We have named this mutant line boygirl (*byg*). Genetic mapping placed *byg* on the proximal region of mouse Chromosome 17 and molecular studies revealed that the *byg* phenotype is caused by a point mutation in the *Map3k4* (*Mekk4*) gene. Embryos doubly heterozygous for both the *Map3k4^byg^* allele and a targeted null allele of *Map3k4* (*Map3k4^tm1Flv^*) exhibited neural tube defects and XY gonadal sex reversal, confirming that *Map3k4* is the causal gene. *Map3k4* encodes a mitogen-activated protein kinase (MAPK) kinase kinase, demonstrating for the first time a role for MAPK signalling in mammalian sex determination. We describe molecular and cellular studies on the *byg* mutant that demonstrate a requirement for mitogen-activated protein kinase kinase kinase 4 (MAP3K4) in regulating XY gonadal growth, mesonephric cell migration, and the expression of *Sry*, and hence *Sox9*, during XY gonad development. We also describe genetic experiments that suggest that loss of *Map3k4* is responsible for a previously reported autosomal sex reversal phenomenon, T-associated sex reversal (*Tas*) [40],[41].

## Results

### Identification and Molecular Characterisation of *byg* Mutant

**Figure 1:**
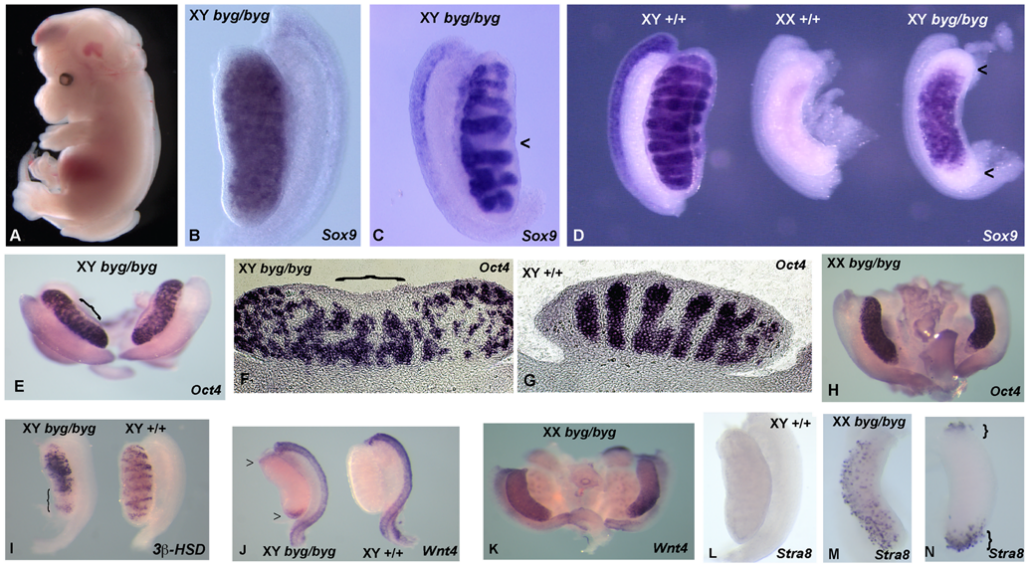
Identification of the *byg* mutant. (A) Line RECB/31 (boygirl, *byg*) mutant embryo dissected during the screen at 13.5 dpc. The embryo shown here has exencephaly, spina bifida, and mild oedema. On the mixed genetic background used for the forward genetic screen, the boygirl XY gonadal phenotypes observed at 13.5/14.5 dpc ranged from testis-shaped gonads that lacked cords (B), to testes with abnormal cord morphology and “missing” cords (arrowhead, C) through to XY gonads with an overt ovarian morphology (right, D). All gonads shown here were hybridised with a *Sox9* probe to assess cord structure and differentiation of Sertoli cells. Note the higher than normal (with respect to ovary development) levels of *Sox9* transcript in the boygirl XY “ovary” in (D) and the absence of *Sox9* from the poles of this gonad (arrowheads, D), a characteristic of ovotestis development. Wild-type littermate gonads from unaffected XY and XX embryos are shown for comparison. (E–G) After further out-crossing of the *byg* mutation to C3H/HeH for mapping purposes, XY *byg/byg* gonads exhibited further signs of ovotestis formation. For example, expression of the germ cell marker *Oct4* at 13.5 dpc revealed a cord-like pattern only in the centre of the gonad shown here (bracket, E), made clearer by sectioning (bracket, F). Note also the thickened capsule over the central testicular area. Control gonads exhibited robust cord formation throughout their length (G). (H) XX *byg/byg* gonads exhibited a normal pattern of *Oct4* expression. (I) The poles of XY *byg/byg* gonads were also characterised by loss of male-specific genes, such as the Leydig cell marker *3β-HSD* (bracket). (J) Abnormal expression of female-specific marker *Wnt4* at the gonadal poles (arrowheads next to gonad on left) at 14.5 dpc. (K) Normal expression of *Wnt4* in XX *byg/byg* gonad. (L) Absence of expression of meiotic marker *Stra8* in wild-type XY gonad. (M) Prominent *Stra8* expression in XX *byg/byg* gonad. (N) Expression of *Stra8* at gonadal poles in XY *byg/byg* gonad (brackets).

Line 31 (RECB/31) was identified in a forward genetic (phenotype-driven) screen for embryonic gonad abnormalities after ENU mutagenesis (see Materials and Methods for details). Embryos homozygous for ENU-derived mutations were isolated and examined for a variety of morphological abnormalities. One RECB/31 embryo, dissected at 13.5 dpc, exhibited spina bifida, mild oedema, and also contained gonads shaped like normal testes but with no visible testis cords (Figure 1A and 1B). A second, independent RECB/31 litter contained an embryo with spina bifida and testes that had fewer cords than normal with an irregular morphology (Figure 1C). Having identified these individuals, subsequent RECB/31 embryos were examined and gonads were collected for sexing and wholemount in situ hybridisation (WMISH). In this manner, another XY individual was identified in which the gonads were morphologically ovarian at the same stage (Figure 1D). WMISH analysis of gonads from these three abnormal embryos using the Sertoli cell marker *Sox9* confirmed the disruption to testis development and its variable severity as described above (Figure 1B–1D). In each case, *Sox9* expression was still prominent. However, in the case of the XY gonad with an ovarian appearance, expression was restricted to the central portions of the gonad and absent from the poles. This observed phenotypic variability, and that of subsequent mutants identified in the RECB/31 pedigree, is likely due to the mixed genetic background of the embryos examined. All embryos with abnormal XY gonads exhibited failure of neural tube closure, either spina bifida or exencephaly (unpublished data). Embryonic death of homozygous mutants was commonly observed after 15.5 dpc. Because of the observed gonadal abnormalities and apparent XY gonadal sex reversal, this mutant line was named boygirl (*byg*).

During subsequent generations of backcrossing onto C3H/HeH the gonadal phenotype was still robust, although the majority of RECB/31 XY gonads had the appearance of ovotestes, in which the central portion of the gonad shows evidence of cord formation, but the poles are ovarian in both appearance and marker expression (Figure 1E–1N). No overt abnormalities were observed in XX *byg/byg* gonads in these marker studies.

**Figure 2:**
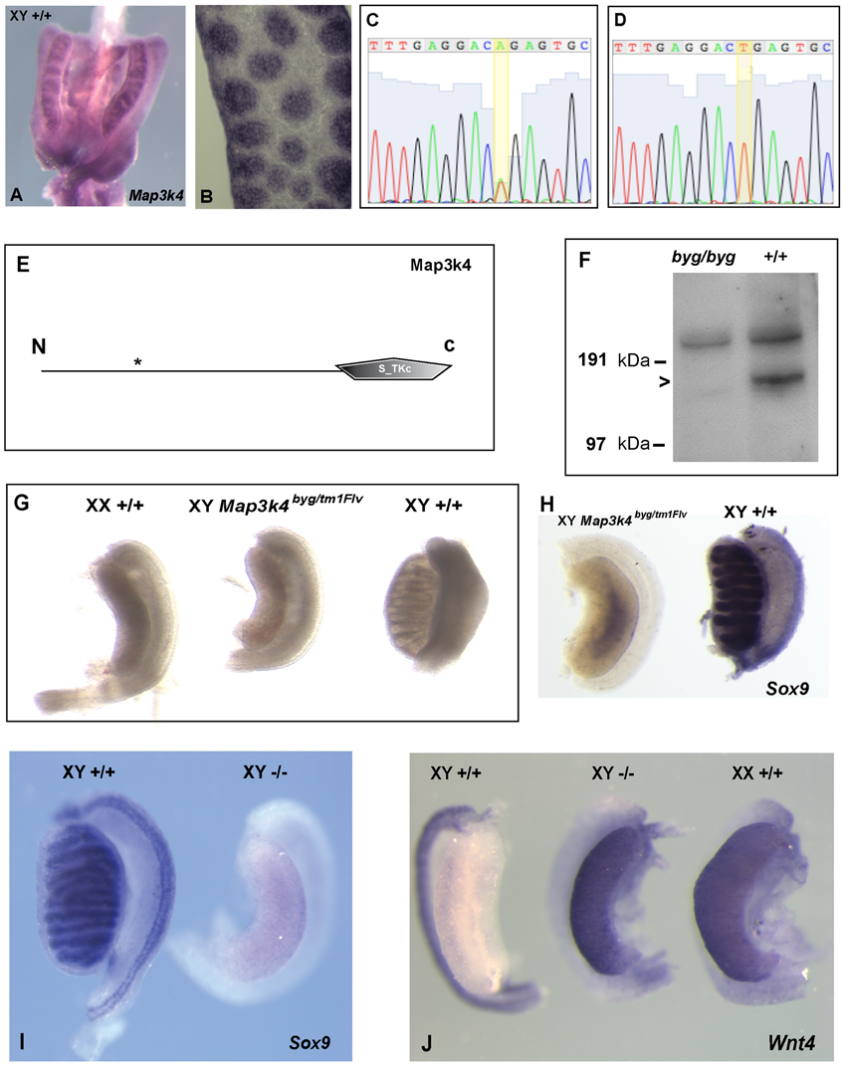
The *byg* phenotype is caused by a point mutation in *Map3k4*. (A) WMISH of 12.5 dpc XY gonads with a *Map3k4* probe revealing widespread expression, including in newly formed testis cords. (B) Longitudinal section through male gonad at 13.5 dpc, after WMISH, showing *Map3k4* expression in testis cords. (C, D) Sequence traces from heterozygous (*byg*/+, C) and homozygous (*byg/byg*, D) individuals reveal an A to T transversion at nucleotide position 1,144 of the *Map3k4* open reading frame of the *byg* allele. (E) This mutation replaces an arginine with a premature stop codon (asterisk) at amino acid position 382 of the 1,597 amino acid MAP3K4 protein. The predicted truncated protein lacks the critical kinase domain (S_TKc) and, therefore, any MAPKKK function. (F) Western blotting of protein extracted from *byg/byg* and +/+ embryos shows absence of full-length (180 kDa) MAP3K4 in mutant homozygotes. The position of size markers is shown on the left. The upper band found in both lanes is due to cross-reaction of the antibody with an unrelated protein. (G) A genetic complementation test was performed to confirm that homozygosity for the *Map3k4* point mutation caused the *byg* gonadal phenotype. XY mutant embryos heterozygous for both the *Map3k4^byg^* and targeted *Map3k4^tm1Flv^* alleles were dissected at 14.5 dpc and contained gonads with an overt ovarian morphology (central gonad). XX and XY littermate controls are also shown. (H) Hybridisation of a *Sox9* probe to a doubly heterozygous XY gonad (left) reveals little expression of the Sertoli cell marker, in contrast to an XY control (right). (I) XY gonad from 14.5 dpc embryo homozygous for the *Map3k4^tm1Flv^* allele (−/−) exhibits overt ovarian morphology and an absence of *Sox9* (right), in contrast to XY littermate control (left). (J) XY gonad from homozygous knockout embryo (−/−) also expresses high levels of *Wnt4* (central gonad). XY and XX control gonads are shown on left and right, respectively.

Identification of additional affected XY gonads permitted mapping of the *byg* mutation. Abnormal embryos (*n* = 9) were typed with a genome-wide panel of 55 SNP markers in order to identify chromosomal regions that were consistently homozygous for the C57BL/6-derived allele. Only one region, on proximal mouse Chromosome 17, showed this feature of genetic association with *byg*. This initial linkage was refined by subsequent backcrossing of *byg* carrier males with C3H/HeH females and intercrossing of carrier progeny, identified by SNP haplotype analysis. Additional SNPs were then used to identify a critical region, in which the *byg* mutation must reside, between 9.66 Mb (rs3665053) and 15.32 Mb (rs13482889) on Chromosome 17. We used an informatics-based approach to identify candidate genes in the *byg* critical region. One such candidate was the gene *Map3k4* (also known as *Mekk4*, GenBank [http://www.ncbi.nlm.nih.gov/Genbank] number NM_011948), which encodes a MAPK kinase kinase [42],[43]. Mice lacking this gene, which were generated previously by gene targeting, are associated with perinatal lethality on the C57BL/6 background [44]. Because homozygous *Map3k4* mutant embryos also exhibit neural tube defects and because *Map3k4* is expressed in most embryonic tissues between 9.5 and 15.5 dpc [42],[44],[45], including the gonads (Figure 2A and 2B), we examined the sequence of *Map3k4* in affected *byg*/*byg* embryos. A single nucleotide substitution at nucleotide position 1,144 of the *Map3k4* open reading frame was identified in the homozygous form in two independent *byg/byg* mutants (Figure 2C and 2D). This substitution replaces an arginine with a premature stop codon at amino acid position 382 of the 1,597 amino acid MAP3K4 protein. The predicted truncated protein lacks the critical kinase domain of MAP3K4 and, therefore, lacks any MAPKKK function (Figure 2E). Absence of full-length (180 kDa) MAP3K4 protein in *byg* homozygous mutants was confirmed by Western blotting with an anti-MAP3K4 antibody (Figure 2F). A kinase-inactive allele of *Map3k4* has previously been shown to have very similar phenotypic consequences to the null allele [45]. Thus, because of the effect of the premature stop codon causing loss of the kinase domain, we conclude that the *Map3k4^byg^* allele is a null allele. The entire colony of *byg* mice was typed for the presence of the mutation in *Map3k4* and all known *byg* carriers were heterozygous for the mutation. The mutation was not found in any wild-type C57BL/6J or C3H/HeH mice. We concluded, therefore, that the gonadal phenotype in mutant *byg* embryos is caused by loss of MAP3K4 function.

To confirm this, and discount the possibility that a second, closely linked mutation in an unrelated gene was responsible for the gonadal phenotype, we studied a second *Map3k4* mutant allele (*Map3k4^tm1Flv^*) generated by gene targeting [44]. Embryos homozygous for the *Map3k4^tm1Flv^* allele exhibit neural tube defects and die perinatally, although there have been no descriptions of sexual development in these individuals. Embryos doubly heterozygous for both *Map3k4^byg^* and *Map3k4^tm1Flv^* were examined at 14.5 dpc and exhibited neural tube defects (unpublished data). All XY embryos contained gonads with an overt ovarian appearance (Figure 2G), and these failed to express *Sox9* at significant levels (Figure 2H). The absence of any overt testicular tissue in these XY embryonic gonads is likely due to the increased contribution from the C57BL/6J genome in these individuals. Embryos homozygous for the targeted allele, which was maintained on the C57BL/6J background, also exhibited gonadal sex reversal, with affected XY embryos containing gonads with ovarian morphology, lacking *Sox9* and expressing *Wnt4*, a marker of ovarian differentiation (Figure 2I and 2J). Thus, these data confirm that *Map3k4* is the gene disrupted in the *byg* mutant and that MAP3K4 is required for testis determination in mice.

### Phenotypic Characterisation of the *byg* Mutant

Evidence exists that the C57BL/6J background is sensitised to disruptions to XY gonad development, and this conclusion appeared to be supported by the increased severity of the XY gonadal phenotype observed in embryos heterozygous for both *Map3k4^byg^* and *Map3k4^tm1Flv^*, and homozygous for *Map3k4^tm1Flv^*, in which the contribution from C57BL6/J was greater. Thus, we performed a detailed examination of embryos homozygous for *Map3k4^byg^* after backcrossing to C57BL6/J for at least two generations. We examined cell proliferation, mesonephric cell migration, and cellular differentiation in mutant and wild-type gonads because all these processes are required for normal testis development [1],[46],[47].

**Figure 3:**
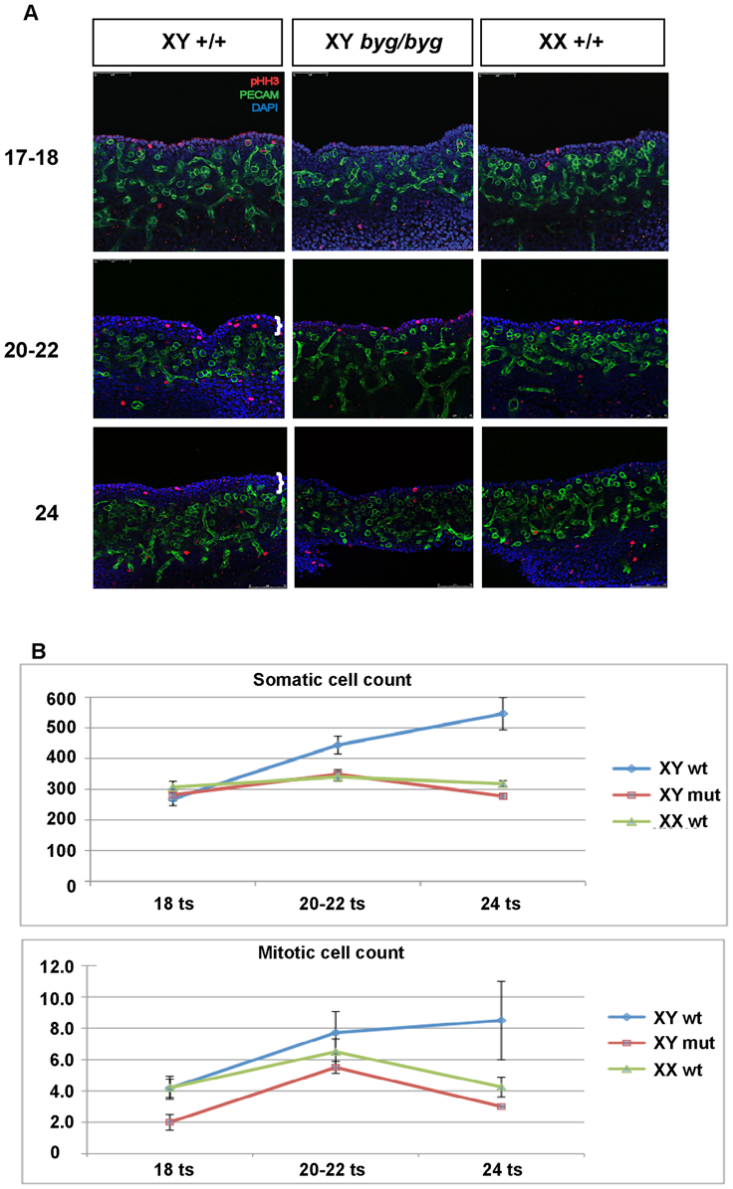
Reduced gonadal growth in XY *byg/byg* embryos between 11.5 and 12.25 dpc. (A) Somatic cell proliferation in the coelomic region of control and XY *byg/byg* gonads was analysed by confocal imaging of wholemount organs after immunostaining with anti-PECAM (green) and anti-phospho-histone H3 (red) antibodies. Cell nuclei were visualised using DAPI staining (blue). All gonads were staged accurately by counting ts (ts number shown on left). The coelomic growth zone characteristic of XY gonads is shown with white brackets in the 22 ts and 24 ts samples. This thickened zone of proliferating somatic cells is not found in XY *byg/byg* or XX +/+ gonads at any of the stages analysed. (B) Counts in the coelomic region of total number of somatic cells and mitotic (pHH3-positive) cells in XX/XY wild-type (wt) and XY *byg/byg* (mut) gonads at the stages shown in (A). Cell counts were performed on limited numbers of XX *byg/byg* gonads at 18 ts and 20–22 ts. Numbers were comparable with wild-type XX and XY *byg/byg* samples (unpublished data). For details of cell counting methodology and statistical tests see Materials and Methods and Table 1.

Cellular proliferation is an important component of the organogenetic programme of testis development [48],[49]. Gonadal cell proliferation was examined at 11.5 dpc (17–18 tail somites [ts]), 12.0 dpc (20–22 ts), and 12.25 (24 ts) in the coelomic region of gonads from *byg/byg* and control littermates using immunostaining with an antibody for the mitotic marker phosphorylated histone H3 (pHH3). Somatic cell proliferation in XY *byg/byg* gonads appeared reduced in the coelomic region in comparison to wild-type XY embryonic gonads at all stages examined (Figure 3; Table 1). Moreover, at the 22- and 24 ts stages (12.0–12.25 dpc), the coelomic region of control XY gonads was thickened and contained a larger number of somatic cells, in contrast to *byg/byg* XY gonads, which had fewer cells in this region and resembled wild-type XX gonads of the same stage (Figure 3). We conclude that cellular proliferation, and thus gonadal growth, in the coelomic region is severely compromised in *byg*/*byg* XY gonads at an early stage.

**Table 1:** Cell proliferation in the XY *byg/byg* gonad.

Genotype	18 ts			20–22 ts			24 ts		
	N	Mitotic	Somatic	N	Mitotic	Somatic	N	Mitotic	Somatic
XY +/+	6	4.17	268.0	7	7.71	444.0	2	8.50	546.0
XY *byg*/*byg*	5	2.00*^a^	281.4	8	5.50*^b^	349.3*^c^	2	3.00	277.0*^d^
XX +/+	5	4.20	306.6	6	6.50	340.0*^e^	4	4.25	317.8*^f^

Cell counts of somatic cells in mitosis (mitotic) and total somatic cells (somatic) in coelomic region of wild-type (+/+) and *byg/byg* gonads between 18 and 24 ts stages. N, independent gonad samples examined.

*, Values are significantly different from respective wild-type values using a two-tailed *t*-test where *p*≤0.05. Individual *p*-values: ^a^0.01; ^b^0.043; ^c^0.001; ^d^0.038; ^e^0.002; ^f^0.003.

Increased levels of apoptosis have previously been described in the neural tube of mice lacking *Map3k4* [44]. For this reason we examined levels of apoptosis in the *byg/byg* XY gonad and controls at 17 ts using an antibody to cleaved caspase 3. We observed very few positive cells in mutant and control gonads, although large numbers of apoptotic cells were observed in a positive control (interdigital mesenchyme of the developing limb) using this assay (unpublished data). Thus, we cannot attribute impaired gonadal growth in XY *byg/byg* embryos to increased levels of apoptosis.

Testis cord formation in the mouse requires cell migration from the associated mesonephros into the XY gonad in a male-specific fashion [50]–[53]. To examine mesonephric cell migration into the XY *byg/byg* gonad we first examined development of mutant gonads when explanted from the embryo at 11.5 dpc and cultured in vitro. Control gonads (wild-type and *byg*/+ littermates) formed clear testis cords after 2 d of culture and expressed the Sertoli cell marker, *Sox9* (*n* = 3) (Figure S1A). In contrast, we did not observe any testis cords in cultured XY gonads from *byg/byg* embryos (*n* = 3) (Figure S1B and S1D). WMISH analysis revealed that these cord-free XY gonads failed to express *Sox9* (Figure S1B), indicating a failure to execute the program of testis differentiation. In contrast, high levels of *Wnt4* expression in the mutant XY gonads indicated an activation of the ovarian pathway (Figure S1D).

To examine whether the severe disruption to cord formation in the *byg/byg* gonad was associated with any loss of mesonephric cell migration, we performed recombination experiments in which mesonephroi ubiquitously expressing green fluorescent protein (GFP) were cultured adjacent to a *byg/byg* XY gonad from 11.5 dpc. Cell migration into control XY gonads was prominent after 48 h of culture (Figure S1E). In contrast, little or no cell migration was detected in cultured *byg/byg* gonads (Figure S1F). These data suggest that two early cellular processes associated specifically with XY gonad development, cell proliferation in the coelomic growth zone and mesonephric cell migration, are disrupted in the absence of MAP3K4.

In order to address the molecular basis of these defects, we next investigated the expression of key male- and female-determining genes and gene-products between 11.5 and 14.5 dpc, stages of gonad development between which the male and female fates are established and the programme of sexually dimorphic morphogenesis is executed. Several molecules have been shown to be required for normal testis determination, including SRY [5], fibroblast growth factor 9 (FGF9) [10], FGFR2 [11],[12], and SRY-like HMG box 9 (SOX9) [7],[8]. Current understanding suggests that SRY, in concert with SF1, acts to up-regulate *Sox9* expression in the XY gonad at 11.5 dpc [54],[55]. *Sox9* expression is then maintained at a high level by a positive feedback loop with FGF9/FGFR2, and acts to antagonise function of the ovary-determining gene *Wnt4* [56]. A role for prostaglandin D2 in the regulation of *Sox9* expression has also been proposed [12],[39],[57],[58]. Downstream of SOX9, genes such as *Amh* [59] and *Vanin-1* [31],[32],[60], with male-determining effects, are transcriptionally activated, and germ cell fate is established by modulation of retinoic acid signalling [36],[37]. These molecular events are associated with precise spatial (cellular and subcellular) and temporal expression profiles of genes and their protein products, often in a sexually dimorphic manner.

**Figure 4:**
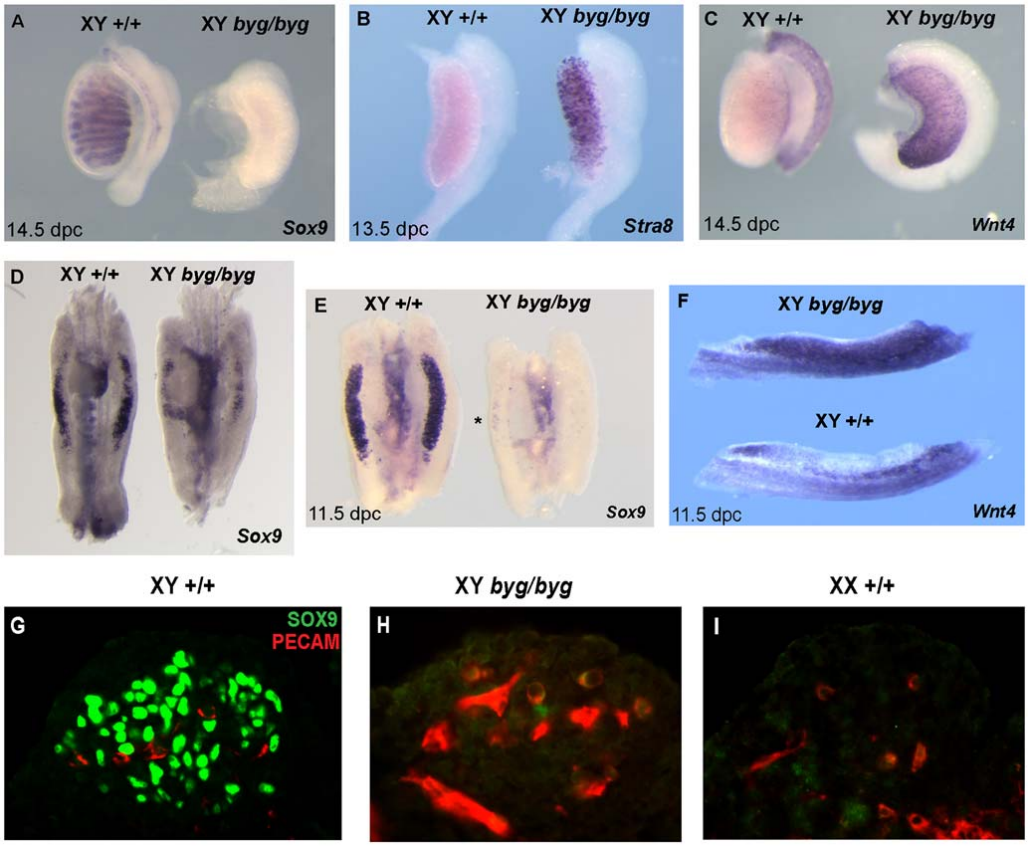
Analysis of *Sox9*, *Wnt4*, and *Stra8* expression reveals gonadal sex reversal in XY *byg/byg* embryos. (A) After back-crossing of the *byg* mutation to C57BL/6J for at least two generations, XY *byg/byg* gonads expressed negligible levels of *Sox9* (left) in comparison to control XY gonads (right) at 14.5 dpc. (B) At 13.5 dpc, XY *byg/byg* gonads express *Stra8*, a marker of meiotic germs cells usually restricted to the ovary. (C) *Wnt4*, a marker of ovarian somatic cells, is expressed in mutant XY gonads (right), in contrast to control gonads (left). (D) On the C3H/HeH background *Sox9* transcription is detected at lower levels in the XY *byg/byg* gonad at 11.5 dpc (right) when compared to wild-type controls (left). Signal in the mutant gonad appears concentrated towards the centre. (E) After backcrossing to C57BL/6J, *Sox9* is expressed abundantly in the XY wild-type gonad at 11.5 dpc (left) but is now absent from XY *byg/byg* gonads, apart from just a few cells visible in the central region (asterisk, right). (F) XY *byg/byg* gonads at 11.5 dpc express the ovarian somatic cell marker *Wnt4* (top), in contrast to an XY control gonad (bottom). (G–I) Immunostaining of a transverse section from a control C57BL/6J XY gonad at 11.5 dpc with anti-PECAM (germ cell, red) and anti-SOX9 (pre-Sertoli cell, green) antibodies reveals large numbers of somatic cell nuclei (G), in contrast to XY *byg/byg* (H), and XX wild-type control (I), in which SOX9 protein is not detected at significant levels.

Given its central role in testis development we began our study with an analysis of *Sox9* expression. From 11.5 dpc onwards *Sox9* transcription in control XY gonads is prominent, initially in pre-Sertoli cells and subsequently in Sertoli cells of the seminiferous cords/tubules. However, analysis of *byg/byg* homozygotes revealed dramatically reduced levels of *Sox9* transcript (Figure 4). At 14.5 dpc the *byg/byg* XY gonad resembles an ovary morphologically and no significant *Sox9* transcription was detectable (Figure 4A). This loss of a Sertoli cell marker in mutant XY gonads was accompanied by elevated expression of two known female-specific markers at the same stage, *Stra8* and *Wnt4* (Figure 4B and 4C). Expression of these genes indicates that the ovarian pathway of development, including entry of germ cells into meiosis, is activated in vivo in the absence of MAP3K4.

At 11.5 dpc, the sex-determining stage of gonadogenesis, little or no *Sox9* transcript was observed (Figure 4E), and this loss of expression was confirmed by immunostaining of mutant and control gonads at the same stage with an anti-SOX9 antibody (Figure 4G–4I). However, *Wnt4* expression was prominent in the XY *byg/byg* gonad at 11.5 dpc, in contrast to wild-type controls (Figure 4F). Interestingly, *Sox9* transcription at 11.5 dpc in mutant gonads on the C3H/HeH background was reduced in comparison to controls (Figure 4D), but not to the same degree as the C57BL/6J-derived mutant gonads at the same stage. Loss of *Sox9* expression is associated with XY sex reversal in a number of genetic contexts, and mice homozygous for a loss-of-function allele of *Sox9* targeted to the developing XY gonads by Cre-mediated excision exhibit immediate, complete gonadal sex reversal, as evidenced by the expression of female-specific markers and the absence of testis cord formation [8]. Thus, loss of *Sox9* expression is sufficient to explain the failure of male-specific events in XY *byg/byg* homozygotes, such as enhanced coelomic region growth, mesonephric cell migration, and testis cord formation.

We next analysed expression of several other important markers of male and female gonad development around 11.5 dpc (16 to 19 ts) using immunohistochemical staining of transverse sections. SF-1 (NR5A-1) is thought to mediate up-regulation of *Sox9* transcription in the early XY gonad by acting on a specific gonadal enhancer (TESCO) in synergy with SRY [55]. We observed no significant difference in the expression of SF-1 between wild-type and *byg/byg* XY gonads at this stage, with large numbers of somatic cells exhibiting nuclear staining in both genotypic classes (Figure S2A and S2B). FGFR2, a gonadal receptor for FGF9, has been reported to exhibit a sexually dimorphic profile of expression in the gonads at 11.5 dpc, with somatic cells in the body of the XY gonad exhibiting nuclear localisation of the protein and XX somatic cells, in contrast, exhibiting a cytoplasmic localisation [12],[61]. We also observed nuclear localisation of FGFR2 in somatic cells of control XY gonads at 11.5 dpc (Figure S2F and S2G), but in XY *byg/byg* gonads, although FGFR2 expression was still prominent, its localisation was cytoplasmic, resembling XX control gonads at the same stage (Figure S2H–S2J).

Next, we examined the early expression of FOXL2, a protein required for normal ovary development [62]–[65]. *Foxl2* transcription has been reported to be up-regulated in the developing mouse gonad around the time of sex determination [66] and restricted to somatic cells [62],[63]. In newborn mice FOXL2 protein is expressed in the nuclei of pregranulosa cells [63]. We observed expression of FOXL2 in the nuclei of somatic cells in wild-type XX gonads at 11.5 dpc (Figure S2E), but negligible expression was observed in wild-type XY gonads (Figure S2C). However, striking up-regulation of FOXL2 was observed in the nuclei of somatic cells of *byg/byg* XY mutants (Figure S2D). Together with prominent expression of *Wnt4* transcript in mutant gonads at the same stage (Figure 4F), these data suggest that the ovarian determining pathway is activated at an early stage in the gonads of XY *byg/byg* embryos lacking MAP3K4.

**Figure 5:**
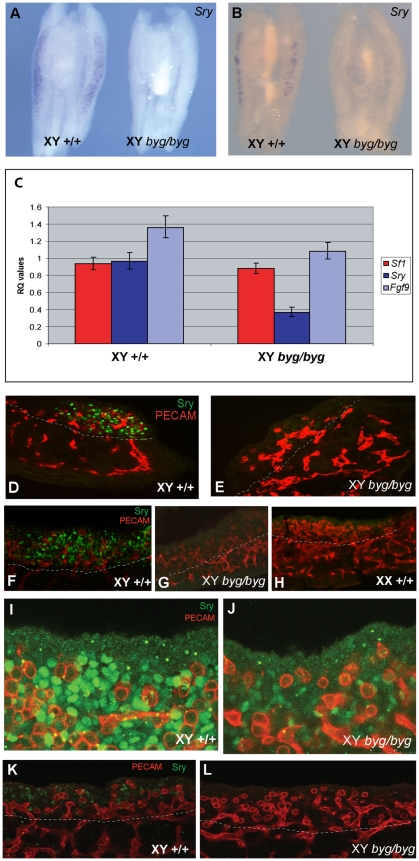
Loss of *Sry* transcript and protein expression in XY *byg/byg* gonads at 11.5 dpc on C57BL/6J. (A) At the 17 ts stage, *Sry* transcript is detected throughout an XY control gonad (left) but is absent from the C57BL/6J XY *byg/byg* gonad (right). (B) By 19 ts, the signal is diminishing in the XY control gonad (left) and is still not detectable in the XY *byg/byg* gonad (right). (C) qRT-PCR analysis of *Sry*, *Sf-1*, and *Fgf9* transcription in XY +/+ and *byg/byg* gonads at 11.5 dpc (17–18 ts). Error bars for the relative quantitation (RQ) values represent variation across four biological replicates for each genotype and three technical replicates for each sample. The 2.8-fold reduction in *Sry* levels in XY *byg/byg* gonads is significant (*p* = 0.02) based on a *t*-test calculated using average dCT values for the above genes and those of *Hprt1*. Differences in the levels of *Sf-1* (*p* = 0.28) and *Fgf9* (*p* = 0.23) were not significant, although there is a trend for reduced expression of *Fgf9* in *byg/byg*. (D) Immunostaining of a longitudinal section of control XY gonad at 17 ts with anti-SRY (green) and anti-PECAM (red) antibodies reveals abundant expression of SRY in somatic cell nuclei of the gonad. Tissue beneath the dotted white line in this and subsequent images is mesonephric. (E) In contrast, very few SRY-positive cells are detectable in a longitudinal section from a XY *byg/byg *gonad at the same stage. (F) Confocal imaging of a control XY gonad after wholemount immunostaining with anti-SRY and anti-PECAM antibodies reveals large numbers of SRY-positive somatic cells, in contrast to XY *byg/byg* (G) and control XX (H) gonads. (I) High magnification confocal image of wild-type gonad at 11.5 dpc showing large numbers of SRY-positive somatic cells (green) and germ cells (red). (J) Confocal image of SRY expression in XY *byg/byg* gonad at 11.5 dpc generated using the same settings as in (I). Note the greatly reduced number of SRY-positive cells and the reduction in signal intensity in the mutant gonad. (K) A wild-type XY gonad at 11.0 dpc (13 ts) showing SRY-positive cells (green) amongst germ cells (red). (L) In contrast, no SRY-positive cells are detected at 11.0 dpc in an XY *byg/byg* gonad.

Absence of a number of molecules has been reported to cause reduction or loss of *Sox9* expression in mutant mouse gonads, including FGF9 [56], FGFR2 [12], WT1 [16],[67], and DAX1 [9],[29]. Recently, it has been shown that SRY and SF-1 cooperatively bind a specific enhancer element (TESCO) to up-regulate *Sox9* transcription during XY gonad development and that SOX9 subsequently acts to maintain its own expression by binding to the same enhancer [55]. Because of this central role for SRY in regulation of *Sox9* expression, we investigated the expression of *Sry* in XY *byg/byg* gonads (Figure 5). *Sry* transcription reaches a peak at 11.5 dpc (17–18 ts) in XY mouse gonads, and so we studied expression at this stage using in situ hybridisation. At 17 ts we observed *Sry* transcripts in wild-type XY gonads using WMISH. However, no significant *Sry* transcription was observed in XY mutant gonads at the same stage (Figure 5A). At the 19 ts stage, *Sry* transcription is reduced in the wild-type gonads and still absent from mutant (Figure 5B). We utilised quantitative real time-PCR (qRT-PCR) to confirm this reduction in *Sry* expression in mutant gonads at 11.5 dpc (Figure 5C). This qRT-PCR study revealed an almost 3-fold reduction in *Sry* transcript levels in XY *byg/byg* gonads. *Sf1* transcript levels did not differ significantly between mutant and control gonads, in line with our immunohistochemistry data. *Fgf9* transcript levels appeared to be reduced in XY *byg/byg* gonads, although this difference was not statistically significant. We then studied the expression of SRY protein in mutant and control gonads at the same stage using a specific antibody to SRY [39],[68]. Expression of SRY was observed in somatic cells of the developing gonad at 11.5 dpc in control XY gonads (Figure 5D and 5F). In contrast, very few SRY-positive cells were detected in XY *byg/byg* gonads, which resembled XX control gonads at the same stage of development (Figure 5E, 5G, and 5H). High magnification examination of XY *byg/byg* gonads at these stages also revealed that those cells that did express SRY did so at a greatly reduced level (Figure 5I and 5J). In contrast to wild-type controls, no SRY-positive cells were detected at 11.0 dpc (Figure 5K and 5L). These studies suggest that appropriate expression of *Sry* in XY gonads, at both the transcript and protein level, is dependent on the presence of MAP3K4. In the absence of MAP3K4, *Sry* expression is delayed and, at 11.5 dpc, severely reduced. Reduced or delayed expression of *Sry* is known to be a cause of XY gonadal sex reversal [69],[70].

### Expression of MAPK Signalling Components in the Developing XY Gonad

**Figure 6:**
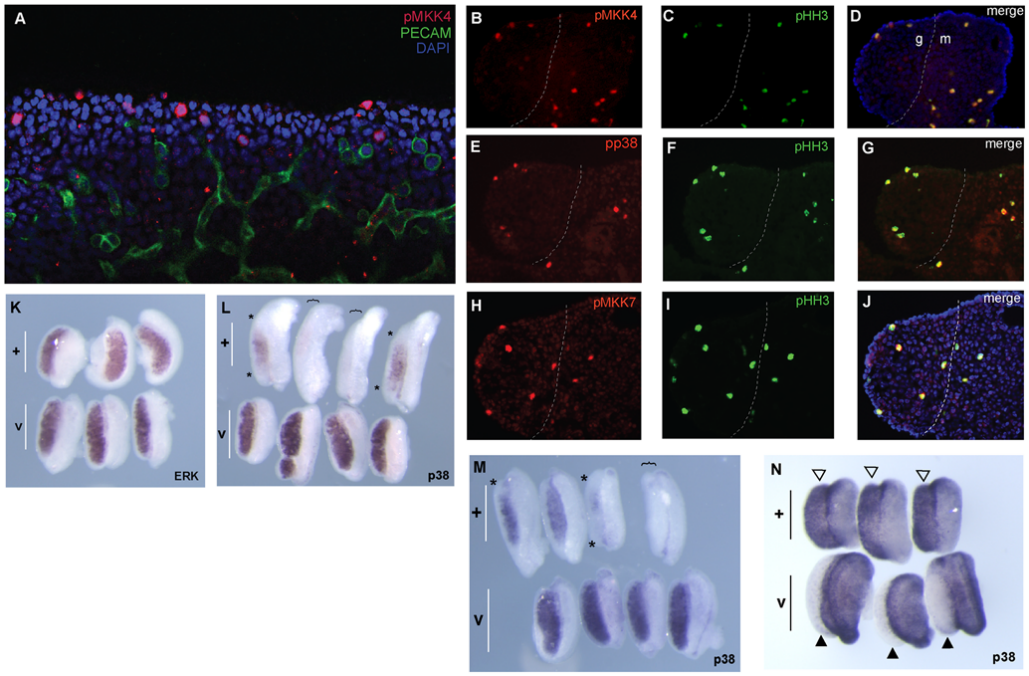
MAPK signalling and XY gonad development. (A–G) Gonadal expression of three activated MAPK signalling components was examined: phospho-MKK4 (pMKK4), a product of MAP3K4-mediated phosphorylation of MKK4, phospho-p38 (pp38), and phospho-MKK7 (pMKK7). (A) Anti-pMKK4 antibody detects activated MKK4 (red) in a number of somatic cells in the developing XY gonad at 11.5 dpc (21 ts) after wholemount immunostaining. Note the concentration of pMKK4-positive somatic cells at the gonadal periphery in the coelomic region. Germ cells are marked by anti-PECAM antibody (green) and cell nuclei by DAPI. (B–D) Transverse sections of 21 ts XY gonad showing co-expression of pMKK4 and the mitotic marker pHH3 in cells at the gonadal periphery. The gonad (left) is separated from the mesonephros (right) by a dotted white line. (E–G) Transverse sections of 21 ts XY gonad revealing co-expression of activated p38 (pp38) and pHH3 in cells at the gonadal periphery. (H–J) Transverse sections showing similar co-expression of pMKK7 and pHH3. (K–N) The effect of two specific inhibitors of MAPK signalling on XY gonad development in vitr*o* was studied. (K) Culture of wild-type XY gonads from 11.5 dpc for 48 h in the presence anti-ERK inhibitor U0126 (+, upper three gonads in panel) has no obvious effect on *Sox9* expression in comparison to gonads cultured with vehicle control (v, lower three gonads). Testis cord formation in treated samples is, however, not as pronounced as in controls. (L, M) Culture of wild-type XY gonads in the presence of p38 inhibitor SB202190 (+, upper rows of gonads) results in striking, but variable, alterations to *Sox9* expression patterns in contrast to vehicle control cultures (v, lower rows), ranging from loss of transcription in the gonadal poles (asterisks) to complete absence of transcription (gonads beneath brackets). (N) Analysis of XY gonads cultured in SB202190 also reveals up-regulation of *Wnt4* transcription (open arrowheads, upper row) in contrast to vehicle controls, which lack *Wnt4* (black arrowheads, lower row). These data suggest that, at least partial, XY gonadal sex reversal is caused by inhibition of p38 from 11.5 dpc. There were no signs of tissue necrosis or excessive cell death during these experiments. All gonads were from embryos on the C57BL/6J background.

MAP3K4 activity results in activation of the p38 and JNK MAPK pathways as part of a three-kinase phosphorelay module [71]. This signalling module is thought to regulate, amongst other things, the cell's response to stress including ultraviolet radiation, heat shock, and osmotic stress [72]. MAP3K4 regulates the MAPKs p38 and JNK via the phosphorylation of the MAP2Ks MKK3/MKK6 and MKK4/MKK7, respectively [42],[43]. A reduction in the number of cells positive for activated MKK4 activity has been reported in the neuroepithelium of embryos lacking MAP3K4 [44]. Therefore, we assayed for the presence of activated MKK4 in wild-type XY gonads at 11.5 dpc using antibodies specific for the phosphorylated form of this protein (pMKK4). pMKK4-positive cells were observed in the gonad, but these were primarily found in the coelomic region of the gonadal periphery (Figure 6A and 6B), a profile reminiscent of pHH3-positive mitotic cells (Figure 3A). A similar distribution was observed when pMKK7-positive cells were imaged (Figure 6H). Given these observations, we assayed directly for co-expression of pMKK4 and pHH3 in the gonad at 11.5 dpc using immunostaining of sections. pMKK4-positive cells were found to be positive for pHH3 too, both in the gonad and adjacent mesonephros (Figure 6B–6D). We then assayed for the presence of activated p38 (pp38) and pMKK7 in the same tissue sections, and observed a similar pattern of pp38- and pMKK7-positive cells at the gonadal periphery, which were also positive for pHH3 (Figure 6E–6J). The co-expression of pMKK4 and pHH3 was also observed in XY *byg/byg* gonads at the same stage. In the case of pMKK4, pMKK7, pp38, and pJNK, cells positive for these activated proteins were still detectable in XY *byg/byg* gonads at 11.5 dpc (Figure S3), consistent with residual pMKK4 expression in the neural tube of embryos lacking MAP3K4 [44]. These data suggest that MAPK signalling is active in the developing XY gonad at early stages, and is associated with proliferating cells of the coelomic growth zone, but that alternative pathways exist for MAPK activation in the gonad in the absence of MAP3K4. Moreover, given our observation that mitotic somatic cells in the coelomic region are those cells with activated MKK4/7 and activated p38, the reduction in the number of proliferative cells in the XY *byg/byg* gonad (Figure 3) corresponds to a reduction in the number of pMKK4/7- and pp38-positive cells. Whilst it is possible that a gonadal somatic cell activates the MAPK pathway only once it enters mitosis, it is more consistent with the known role of MAPK signalling in cell proliferation to conclude that male-enhanced proliferation in the coelomic region is a MAPK-dependent process. The reduction of coelomic region growth in the XY *byg/byg* gonad at 11.5 dpc is thus explicable by a reduction in the number of cells exhibiting MAP3K4-mediated phosphorylation of MKK4/7, p38, and possibly other MAPK signalling components.

### Inhibition of p38 MAPK Signalling Disrupts Testis Development In Vitro

In order to address whether disruption to components of MAPK signalling can disrupt testis development in vitro, we cultured wild-type embryonic XY gonads from 11.5 dpc for 48 h in the presence of highly selective inhibitors of the MAPKs extracellular signal-related kinase (ERK) (U0126) and p38 (SB202190) [73]. We then assayed for the expression of *Sox9* using WMISH (Figure 6K–6M). Similar experiments to address the role of JNK were not performed because of the unavailability of highly specific small molecule inhibitors. We observed little affect on *Sox9* expression in gonads treated with ERK inhibitor when assayed by WMISH, although testis cord formation did not occur in treated samples with the same efficiency as samples cultured in vehicle control (Figure 6K). These data are consistent with other reports that MEK1/ERK inhibitors fail to significantly disrupt testis development in vitro [74]. In contrast, culturing in the presence of p38 inhibitor resulted in dramatic reduction of *Sox9* expression, including an almost complete loss of signal in 50% of treated samples (*n* = 8) (Figure 6L and 6M). Examination of *Wnt4* expression in SB202190-treated cultured explants (*n* = 3) also revealed robust expression of this ovarian marker in contrast to vehicle controls (Figure 6N), suggesting that at least partial gonadal sex reversal was occurring during culture of XY explants because of abrogation of p38 activity. In this context, it is interesting to note that human SRY has been recently identified as a possible target of p38 MAPK signalling in cultured keratinocytes [75].

### FGF9-Mediated Activation of *Sox9* Transcription Is Not Abrogated In *byg/byg* Gonads

**Figure 7:**
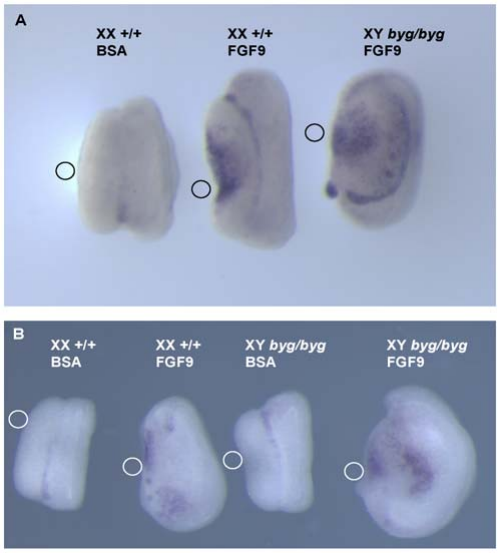
Exogenous FGF9 can activate *Sox9* transcription in gonads lacking functional MAP3K4. (A) Culture of a wild-type XX gonad (left) from 11.5 dpc for 48 h in the presence of a bead coated in BSA does not activate *Sox9* transcription based on in situ hybridisation analysis. In contrast, a bead coated in FGF9 does result in up-regulation of *Sox9* in both XX control gonads (centre) and XY *byg/byg* gonads (right). The small circle adjacent to each gonad shows the approximate position of the bead during culture. (B) In a separate experiment, FGF9-coated beads again activate *Sox9* transcription in wild-type XX and XY *byg/byg* gonads, but a bead coated in BSA does not activate *Sox9* transcription in XY *byg/byg* gonads. Note the increased size of gonads, both XX and XY, after exposure to exogenous FGF9. All gonads were from embryos on the C57BL/6J background.

Given the importance of two components of the FGF signalling pathway, FGF9 and FGFR2, in XY gonad development, we next studied whether *byg/byg* gonads exhibited defects in this pathway by determining whether FGF9 was able to activate *Sox9* transcription in XY *byg/byg* gonads. It has previously been reported that FGF9 is capable of activating *Sox9* transcription in developing XX gonads if they are cultured in the presence of beads coated in this growth factor [56]. In an attempt to address the question of which upstream, extracellular signals employ MAP3K4-dependent phosphorylation during XY gonad development, we determined whether FGF9-mediated activation of *Sox9* transcription was abrogated in MAP3K4-deficient gonads. XY gonads from *byg/byg* and control embryos were cultured at 11.5 dpc for 48 h in the presence of FGF9-coated beads (or beads coated in bovine serum albumin [BSA]) and were then analysed for the presence of *Sox9* transcripts in cells contacting the bead using in situ hybridisation. BSA-coated beads did not induce *Sox9* transcription in any samples. In contrast, *Sox9* transcripts were clearly detected in the vicinity of beads in both cultured wild-type XX gonads and in XY *byg/byg* gonads (Figure 7A and 7B). These data suggest that MAP3K4 is not an obligatory component of signal transduction pathways employed by FGF9 to activate transcription of *Sox9* in the developing gonad. However, failure of normal SRY, and thus SOX9, expression in *byg/byg* XY gonads may result in failure to establish the positive feedback loop between SOX9 and FGF9/FGFR2 [56].

### *Map3k4* Haploinsufficiency Contributes to *Tas*

A locus on mouse Chromosome 17 associated with XY sex reversal and ovotestis formation has previously been described [40]. This mutation, known as *Tas*, was identified in a mouse stock carrying the hairpin-tail (*T^hp^*) deletion whilst being crossed to the C57BL/6J background. The presence of an AKR/J-derived Y chromosome is also required for the development of ovarian tissue in XY C57BL/6J *T^hp^*/+ individuals. It has been hypothesized that the *Tas* mutation resides within the region of the *t* complex deleted in *T^hp^* and hemizygosity for the relevant locus causes varying degrees of sex reversal when on the C57BL/6J Y^AKR^ background [41]. This genetic background is known to be very sensitive to disturbances in the early events of testis development induced by gene mutation [14], and thus one potential explanation for the *Tas* phenotype is haploinsufficiency for a *t* complex locus that is ordinarily testis determining. *Map3k4* is located on proximal mouse Chromosome 17 in the region of the t complex and, in the form of the previously anonymous DNA marker *D17Rp17* (still a synonym of *Map3k4*, see http://www.informatics.jax.org/searches/accession_report.cgi?id=MGI%3A1346875 and GenBank entry NM_011948), has been shown to map within the *T^hp^* deletion [76]. Given this map position and the gonadal phenotype of XY embryos lacking functional *Map3k4* on C57BL/6J, we hypothesized that haploinsufficiency for this gene might be, at least partially, responsible for the *Tas* gonadal sex reversal phenotype.

**Figure 8:**
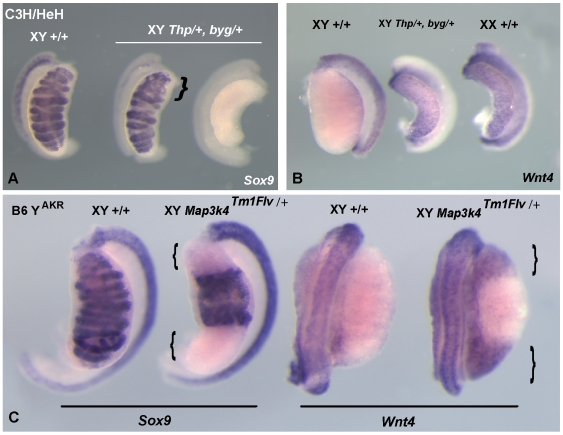
Hemizygosity for *Map3k4* contributes to *Tas*. (A, B) A genetic complementation test demonstrates that *Map3k4* resides in the *Thp* deletion of proximal mouse Chromosome 17. XY embryos doubly heterozygous for the *Map3k4^byg^* allele and the *Thp* deletion (XY *Thp*/+, *byg*/+), on the C3H/HeH background, exhibit a range of defects of gonad development similar to *byg/byg* homozygotes, including testes with regions lacking clear cord morphology (bracket, A) and XY gonads with an overt ovarian appearance that lack *Sox9* transcription (right-hand side gonad, A). Sex-reversed XY gonads express *Wnt4*, a marker of ovary development (central gonad, B), in contrast to XY controls. (C) Ovotestis development in embryos heterozygous for the *Map3k4^tm1Flv^* knockout allele, on the C57BL/6J XY^AKR^ background (B6Y^AKR^), characterised by gonadal poles (brackets) lacking markers of testis development (*Sox9*), and expressing markers of ovary development (*Wnt4*).

We tested this model in two ways. First, we generated embryos doubly heterozygous for the *byg* mutation and the *T^hp^* deletion. If *Map3k4* resides within the *T^hp^* deletion these embryos will lack *Map3k4* function because of failure of complementation and will recapitulate the phenotype of *byg/byg* homozygous embryos. Figure 8 shows that XY *byg*/+, *T^hp^*/+ embryos exhibited abnormalities of testis development. XY gonads dissected from doubly heterozygous embryos at 13.5/14.5 dpc showed disruption to cord morphology or gonadal sex reversal, in which *Sox9* transcription is lost (Figure 8A) and *Wnt4* transcription is activated (Figure 8B). Doubly heterozygous mutants also exhibited neural tube defects (unpublished data). We performed this cross on the C3H/HeH background because this strain has not previously been associated with sensitisation to events disrupting testis development, even given the presence of the Y^AKR^ chromosome [41]. We confirmed, therefore, that *Map3k4* resides in the *T^hp^* deletion and that this deletion, combined with a loss-of-function allele of *Map3k4*, causes varying degrees of disruption to XY gonad development even in the absence of any other predisposing genetic factors.

Secondly, we performed a cross to test directly whether *Map3k4* haploinsufficiency might account for the development of ovarian tissue in XY^AKR^ *T^hp^*/+ C57BL/6J individuals. We generated XY^AKR^ mice after backcrossing of Y^AKR^ to C57BL/6J for six generations. These males were then crossed with females heterozygous for the targeted null allele of *Map3k4* (*Map3k4^tm1Flv^*), also on C57BL/6J, to generate XY^AKR^ *Map3k4^tm1Flv^*/+ heterozygotes on a C57BL/6J background. Nine of these individuals were generated in five litters and seven were scored as normal males based on examination of the external genitalia. However, two were scored as phenotypic females on the basis of external genitalia morphology. Examination of these sex-reversed individuals revealed the presence of ovaries and uterine horns. Histological examination of the ovaries from one of these individuals showed them to be smaller than controls and lacking clearly discernible follicles or ova (unpublished data). Examination of four other heterozygous males at approximately 11 wk of age revealed that these had testes of reduced size (ranging from 0.03 g to 0.08 g, mean = 0.06 g±0.015), in contrast to wild-type controls (*n* = 6, ranging from 0.08 g to 0.11 g, mean = 0.093 g±0.009). Small testes are sometimes an indication of earlier ovotestis development. To test this possibility, we performed timed matings in order to examine gonadal morphology in XY^AKR^ *Map3k4^tm1Flv^*/+ embryos at 14.5 dpc. Of four XY^AKR^ *Map3k4^tm1Flv^*/+ embryos examined, one contained gonads with an overt ovarian morphology, whilst three contained ovotestes identified by morphology and the familiar variegated expression of *Sox9* and *Wnt4* (Figure 8C). On the basis of the XY gonadal sex reversal, complete and partial, observed in adult and embryonic *Map3k4^tm1Flv^*/+ individuals on C57BL/6J-XY^AKR^, we conclude that haploinsufficiency for *Map3k4* is a major contributory factor to male-to-female sex reversal observed in XY^AKR^ C57BL/6J *T^hp^*/+ individuals.

## Discussion

Here we describe evidence demonstrating, for the first time to our knowledge, an in vivo role for the phylogenetically ancient MAPK signalling cascade in mammalian sex determination. XY embryos lacking functional MAP3K4 on a predominantly C57BL/6J background exhibit embryonic gonadal sex reversal associated with failure of a number of cellular and molecular events, paramount amongst these being failure to transcriptionally up-regulate *Sry* and, presumably as a consequence, *Sox9* in the developing gonad at 11.5 dpc. Previous studies, often involving analyses of *Mus domesticus*-derived *Sry* alleles on a C57BL/6 background, have suggested that the testis determining pathway is exquisitely sensitive to levels and timing of *Sry*: if a threshold level is not met in a critical time window, ovary development is likely to ensue [69],[70],[77]. Thus, attention is naturally focussed on the possible explanation for reduced *Sry* expression, at the transcript and protein levels, in XY *byg/byg* gonads. Three potential explanations exist: (i) that a transcriptional regulator (or regulators) required for transcription of *Sry* in pre-Sertoli cells does not function appropriately because of, either directly or indirectly, the absence of MAP3K4-mediated signalling; (ii) that insufficient numbers of pre-Sertoli cells are established in the XY *byg/byg* gonad; (iii) a combination of both of the above effects. With respect to the second hypothesis, the coelomic epithelium is thought to be a source of pre-Sertoli cells in the early XY gonad (prior to 11.5 dpc) [78]. Thus, the reduction in cell proliferation and gonadal growth in the coelomic region of XY *byg/byg* mutant embryos might be considered evidence of a wider range of defects in the developmental potential of the mutant coelomic epithelium and associated mesenchyme, perhaps extending to a reduction in the provision of pre-Sertoli cells, or the provision of pre-Sertoli cells competent to activate transcription of *Sry*. This hypothesis is consistent with the active MAPK signalling that we report in the coelomic region at 11.5 dpc in XY gonad. However, it should be noted that in other genetic contexts in which cell proliferation in the coelomic region of the developing XY gonad is disrupted, such as in gonads lacking *Fgf9* [10],[61], *Sry* transcription is reported to be unaffected [56]. Thus, there is no established mechanistic link between prior proliferative defects in the early gonad and subsequent loss of *Sry* expression. However, given the reported role of FGF9 in promoting gonadal cell proliferation [61], it is possible that loss of MAP3K4 results in an inability of coelomic region cells to efficiently transduce FGF9 signal produced by initial SRY-positive pre-Sertoli cells. This, in turn, would result in failure to establish a positive feedback mechanism by which cell proliferation and SRY expression mutually promote each other, causing insufficient provision of pre-Sertoli cells. This model would explain the reduced numbers of SRY-positive cells detected in XY *byg/byg* gonads between 11.0 and 11.5 dpc (Figure 5). In order to establish whether there is a paucity of cells migrating into the XY *byg/byg* gonad at around 11.2–11.4 dpc to populate the pre-Sertoli cell niche, it will be necessary to perform single-cell labelling experiments similar to those used to establish the role of the coelomic epithelium in this process [78]. However, establishing whether a marked cell was undergoing, or had undergone, active MAPK signalling of the appropriate sort would be technically daunting.

With respect to the first hypothesis, little is known about the transcriptional control of *Sry*, although several potential activators have been described including M33, WT1(+KTS), GATA4/FOG2, and SF1 [79]. This hypothesis is supported by the presence of a few SRY-positive cells in the XY *byg/byg* gonad at 11.5 dpc that exhibit a significant reduction in the intensity of the SRY signal, and also the existence of FOXL2-positive cells in the XY *byg/byg* gonad at 11.5 dpc, since this lineage is arguably the ovarian equivalent of the pre-Sertoli cell lineage of the testis. Evidence already exists for MAPK-dependent phosphorylation of SF1 [80],[81] and GATA4 [82] in other contexts, as a means of increasing their transcriptional activation potency. It is also noteworthy that SRY, which is phosphorylated in humans [83], has recently itself been proposed to be a target of p38-mediated signalling pathways on the basis of cell line studies in vitro [75]. We are currently attempting to identify reduced phosphorylation of candidate testis-determining proteins in MAP3K4-deficient embryonic gonads. However, we cannot rule out the possibility that previously uncharacterised molecules are the key effectors of MAPK-mediated events during gonadogenesis. Moreover, MAP3K4-mediated events required for normal *Sry* transcription may occur in the progenitors of pre-Sertoli cells, in the form of programming, rather than pre-Sertoli cells themselves. In conclusion, the data suggest that the third hypothesis may best explain the observations concerning SRY expression.

The similarity in the phenotypes of mice lacking the *Map3k4* gene [44] and those merely lacking a functional kinase domain of the same gene [45], strongly argues that MAP3K4 functions primarily to regulate MAPK signalling through its kinase domain. Thus, although we cannot formally exclude additional functions, we conclude that loss of functional MAP3K4 in the *byg* mutant results in disrupted MAPK signalling during gonad development. Although ours is the first report of a requirement for MAPK signalling in sex determination in vivo, one previous report has implicated a MAPK scaffolding protein, Vinexin-γ, in regulation of *Sox9* transcription during gonad development [84]. However, the fetal gonads of both XX and XY embryos lacking Vinexin-γ are morphologically normal and adult mice of the same genotypes are viable and fertile. Moreover, *Sox9* transcript levels in *Vinexin*-γ *−*/*−* XY gonads at 12.5 dpc are 75% that of *Vinexin*-γ *−*/+ gonads, suggesting that any modulation of *Sox9* transcription by Vinexin-γ is relatively modest. These data appear to be consistent with reported organ culture studies in which the MAPK inhibitor PD98059 did not significantly inhibit testis cord formation in XY gonad explants [74]. In contrast to the Vinexin-γ studies, we observe an almost complete absence of *Sox9* at the sex determining stage of gonad development (11.5 dpc) in C57BL/6J XY embryos lacking MAP3K4 and a complete failure of testis cord formation at later stages.

One possible explanation of the apparent discrepancy in these observations with respect to the role of MAPK signalling in testis development is the focus in other studies on the MEK-ERK pathway of MAPK signalling, sometimes called the classical MAPK cascade [73]. It has been proposed that Vinexin-γ mediates its effects on *Sox9* transcription in vitro via male-specific activation of the MAPK, ERK [84], and PD98059 is a specific MEK-ERK inhibitor [73],[85]. The focus on MEK-ERK in other studies is likely a consequence of the inviting similarities between requirements for *Sox9* up-regulation during gonad development and chondrogenesis. FGF-mediated activation of *Sox9* transcription during chondrogenesis has been shown to be blocked by the MAPK inhibitor U0126 [86]. U0126 is also a specific MEK-ERK inhibitor [73],[85]. Given that MAP3K4 is thought to act ultimately by activation of the MAPKs p38 and JNK [42],[43], the focus on ERK activation and the consequences of its disruption as a means of determining the role of MAPK signalling during gonad development may have been overly restrictive and resulted in misleading conclusions. Our studies utilising specific small molecule inhibitors of MAPK signalling in organ culture assays corroborate previous observations that MEK-ERK inhibition does not significantly disrupt *Sox9* expression in vitro. However, in contrast, they do suggest a possible role for p38 in gonadal *Sox9* transcriptional regulation and testis cord formation. The significance of these in vitro observations for the possible role of p38 in the aberrant phenotype of the MAP3K4-deficient gonad is unclear, given that *Sry* transcription is already at its peak at 11.5 dpc, the approximate stage at which gonadal explants were employed for in vitro culture experiments. Inhibition of p38 at these stages may disrupt testis-determining events downstream of regulation of *Sry* transcription, perhaps related to regulation of *Sox9* expression, in a manner analogous to that reported for the IL-1β-dependent induction of *SOX9* expression in human articular chondrocytes [87], or disruption of SOX9 function itself. Mice constitutively lacking the alpha isoform of p38 die at around 10.5 dpc, before gonadogenesis can be fully examined [88]. For this reason, it is important to remain open-minded about how many distinct steps in testis development require MAPK-dependent events. Teasing these out genetically will require a conditional null allele of *Map3k4* (and genes encoding other MAPK signalling elements) and inducible, cell-type-specific Cre lines. It will also be important to determine whether disruption to individual MAP2Ks and MAPKs also results in abnormal gonad development in vivo, or whether loss of MAP3K function is disruptive to a broader range of MAPK signalling events, including potential compensatory ones, and thus more likely to result in phenotypic abnormalities.

In addition to downstream events mediated by MAP3K4, it is not yet clear which upstream signals employ MAP3K4 for their transduction. Analogies with chondrogenesis, as described above, have tended to focus attention on the role of FGF signalling and its use of MAPK for its transduction. Moreover, FGF9 is known to be required for the male-specific elevated proliferation rate in the gonadal coelomic region at around 11.5 dpc [61]. However, we have demonstrated that the ability of exogenous FGF9 to activate *Sox9* transcription during gonad culture remains unaltered in the absence of MAP3K4. These data do not definitively demonstrate that FGF9 does not employ MAP3K4-mediated signal transduction during regulation of *Sox9* expression during male gonad development in vivo, but they do suggest that such a pathway is not obligatory. Moreover, initial up-regulation of *Sox9* transcription, along with *Sry* transcription, proceeds as normal in embryonic gonads lacking FGF9 [56]. It is, rather, the maintenance phase of *Sox9* transcription in developing male gonads that is disrupted in the absence of FGF9. Taken together, these observations suggest that we should look at other pathways, in addition to FGF, for the activating signals that require MAP3K4 for their transduction. Although activation of MAPK is a widespread phenomenon, ligand binding to receptor tyrosine kinases (RTK) is commonly associated with activation of this intracellular signalling cascade [89]. Interestingly, the insulin receptor tyrosine kinase gene family (*Ir*, *Igf1r*, and *Irr*) has previously been shown to be required for testis determination through its regulation of *Sry* expression [17], and a number of reports describe a requirement for MAPK in signal transduction through this family of receptors in different biological contexts [90],[91]. Similarly, loss of another RTK, PDGFRα, also disrupts testis development [21] and PDGF signalling is reported to employ MAPK [92]. Finally, in addition to RTK activity, prostaglandin D2 (PGD2) has been shown to influence Sertoli cell differentiation and SOX9 activity [39],[57],[58], presumably through its G-protein coupled receptors, DP and CRTH2 [93], although this is not established. PGD2 signalling in other contexts has been shown to require MAPK [94],[95]. Although the details of MAPK activation in these disparate systems vary, they are all potentially relevant to the phenotype of MAP3K4-deficient gonads because evidence suggests cross-talk between distinct MAPK pathways [75].

Despite the above observations, we cannot rule out the possibility of a role for a hitherto unrecognised growth factor or other extracellular signal in the employment of MAP3K4 during testis development. One virtue of invoking a requirement for MAP3K4 in FGF9-mediated signalling during gonadogenesis in vivo is that this model does not predict a requirement for sexually dimorphic expression of MAP3K4, consistent with *Map3k4* expression data. We observed near ubiquitous expression of *Map3k4*, including male and female gonads, although higher levels were detected in particular cell types. Because of a lack of the relevant antibodies, we were unable to assay for the presence of activated MAP3K4 specifically in XY gonads, although such activation is predicted by the existence of MAP4Ks [96]. It should also be noted that the same explanatory virtue applies to invoking a requirement for MAP3K4 in activation of *Sry* transcription.

We also report here data indicating that haploinsufficiency for *Map3k4* is sufficient to account for *Tas* [40], a phenomenon that has remained unexplained at the molecular level since its discovery more than 20 y ago. XY embryos heterozygous for the *Map3k4^tm1Flv^* mutation on the C57BL/6J-Y^AKR^ background exhibit testicular abnormalities, including XY ovary and ovotestis development, reminiscent of XY^AKR^ C57BL/6J *T^hp^*/+ embryos [40]. Moreover, two adult XY *Map3k4^tm1Flv^*/+ individuals developed as phenotypic females, and both contained ovaries. Four others exhibited testicular hypoplasia, which is associated with prior ovotestis development. It is unclear, however, despite the role for *Map3k4* haploinsufficiency established here, whether additional testis-determining genes exist in the region deleted in *T^hp^*, or whether chromosome deletions themselves predispose XY embryos to sex reversal by inhibitory effects on fetal growth [97].

Significantly, it has been demonstrated that, on the appropriate genetic background, the loss of a single copy of a male-determining gene can result in XY gonadal sex reversal [14]. It has been proposed that such phenotypic effects in mice caused by a single disrupted allele mimic the more common situation in humans, where loss of a single functional copy of genes such as *SF1*, *SOX9*, or *WT1* can result in the development of XY females [98],[99]. Our findings suggest that the loss of a single copy of *Map3k4*, caused by the *T^hp^* deletion or targeted gene deletion, is another example of such a case. Thus, we propose that haploinsufficiency of *MAP3K4* could be the cause of previously unassigned cases of XY gonadal dysgenesis in humans [100]. A second, independent case of *Tas* on the C57BL/6J XY^AKR^ background (B6-TAS) is caused by the T-Orleans deletion (*T^Orl^*), which overlaps with the hairpin tail deletion and also includes *Map3k4/D17Rp17* [41]. Interestingly, it has been proposed that B6-TAS in *T^Orl^*/+ XY^AKR^ mice is due to biologically insufficient levels of *Sry* expression [101]. An analogous explanation of the mechanism underlying the *T^hp^*/+ XY^AKR^ phenotype is consistent with the report here of delayed and reduced levels of *Sry* transcription in *byg/byg* XY gonads at 11.5 dpc. Levels of *Sry* transcription in XY embryonic gonads of *Map3k4^byg/+^* or *Map3k4^tm1Flv/+^* heterozygotes on the C57BL/6J-Y^AKR^ background have yet to be determined, but this experiment will form part of a more extensive analysis of gonadogenesis in these individuals.

Our data have opened a novel entry point into the molecular genetic control of mammalian sex determination and, in particular, the regulation of *Sry* expression. We know of no other higher organisms in which MAPK signalling is thought to regulate sexual development, although pheromone response during mating in yeast and other fungi is known to require a highly related pathway of kinase activity [102]–[104]. We are currently investigating the role of other proteins required for MAPK signalling in mouse gonad development, utilising in vivo and in vitro methods. The ultimate aim of these studies is to clarify the pathway of MAPK signalling that operates during gonadogenesis and determine precisely how it interacts with the molecular events constituting sex determination. Finally, our study suggests that forward genetic screens in the mouse should be considered as another important tool for identifying vertebrate sex determining genes.

## Materials and Methods

### Forward Genetic Screen, Mouse Breeding, and Embryo Generation

We have previously described the mutagenesis and screening methodology employed here [105]. Briefly, a three-generation (G3) recessive mutagenesis screen was used in which C57BL/6J males were injected with ENU and outcrossed to C3H/HeH females; F1 (founder) males were used to establish pedigrees by mating to C3H/HeH and F2 female offspring were backcrossed to their father. Using this breeding scheme it is expected that approximately one in eight embryos in a pedigree will be homozygous for any given ENU-induced mutation. G3 embryos were examined at 13.5 and 14.5 dpc for developmental abnormalities. Examination of pedigree RECB/31 (*byg*) revealed several embryos with abnormal male gonad development. Affected embryos were used for genetic mapping with a 55-marker genome wide SNP panel (sequences available on request). *byg* was maintained by backcrossing to C3H/HeH and, following identification of the *Map3k4* mutation, genotyped for the mutant SNP by pyrosequencing. Timed matings were used to generate embryos at specific stages. Breeding pairs were set up at approximately 3 pm and vaginal plugs were checked the following morning. Noon on the day of the plug was counted as 0.5 dpc. Embryos were typed for chromosomal sex as previously described [106].

Genotyping for the *byg* mutation was performed using a PCR-based pyrosequencing assay using the following primers: Forward PCR primer: 5′-AGGACTATGAACGGTACGC-3′; Reverse PCR primer: 5′-Bio-CGCAGCTTCTGATTTAGATC-3′; Sequencing primer 5′-GCCAAGGACTTTGAGG-3′. *byg* was backcrossed to C3H/HeH and C57BL/6J. Analysis of *byg/byg* embryos on C57BL/6J was performed between generations *n* = 2 to *n* = 5.

The generation and maintenance of mice lacking *Map3k4* has been previously described [44],[107]. *Map3k4-*deficient mice utilised here were maintained on a C57BL/6J background. Hairpin tail (*T^hp^*) mice, originally archived on a mixed genetic background, were rederived using independent in vitro fertilisation (IVF) with both C57BL6/J and C3H/HeH oocytes. *T^hp^* was maintained on both C57BL/6J and C3H/HeH. XY sex reversal was observed on the former, but not the latter, genetic background. *T^hp^* carriers were identified by the shortened tail [108]. Confirmation of the presence of the AKR-derived Y chromosome was performed by using a PCR assay based on that described in [109], which exploits a *Zfy-2* polymorphism between *M. domesticus* and *M. musculus*.

### Expression Studies

WMISH to explanted gonads was performed as previously described [31],[106]. The following probes were used for WMISH: *Sox9* [110]; *Oct4* [111]; *3*β*-HSD* [112]; *Wnt4* (IMAGE clone 40044945), *Sry* [113], *Stra8* (IMAGE clone 40045823), *Map3k4* (IMAGE clone 5705378).

### qRT-PCR

Total RNA and protein were extracted from individual 11.5 dpc (17–18 ts) mouse urogenital ridges (comprising gonad and mesonephros) using the Nucleospin RNA/protein isolation kit (MACHEREY-NAGEL) following manufacturer's instructions. The quantity and quality of the RNA was assessed using the Nanodrop ND1000 (Isogen Life Science) and by gel electrophoresis. A two-step real-time analysis approach was taken. First, cDNA was synthesised using the AB High Capacity cDNA Reverse Transcription Kit using 1 µg of total RNA. The following TaqMan assays (Applied Biosystems [AB]) were used: *Sf1* (Mm00496060_m1); *Fgf9* (Mm00442795_m1); *Sry* (Mm00441712_s1); *Hprt1* (Mm01545399_m1). For each assay, reactions were performed in triplicate using AB Fast Mastermix in a final volume of 20 µl (5 µg of cDNA added). Real-time amplification was performed on an AB 7500 Fast machine, using the manufacturer's recommended program for Fast Mastermix. Analysis of the results was performed using AB software, employing a ddCt method with the gene *Hprt1* as the endogenous control. For each assay four biological replicates and three technical replicates were performed. Statistical analysis was performed using a non-paired *t*-test on the average dCt values calculated for the three technical replicates of each independent sample (biological replicate).

### Immunohistochemistry and Confocal Imaging

The following antibodies were used in this study: SRY [39]; SOX9 [39]; FGFR2 (Santa Cruz number sc-122); SF1, a kind gift from K. Morohashi; FOXL2: antibodies were raised in rabbits against the peptides MMASYPEPEDAAGAALL and WDHDSKTGALHSRLDL, previously utilised in [114]. Antibodies were affinity purified and tested prior to use: platelet/endothelial cell adhesion molecule (PECAM) (BD Bioscience number 553708); phospho-histone H3 (pHH3, Sigma number HH908 or Upstate number 06-570); phospho-MKK4 (Cell Signalling number 9151); phospho-MKK7 (Cell Signalling number 4171); phospho-p38 (Cell Signalling number 4631); phospho-JNK/SAPK (Cell Signalling number 9251); cleaved caspase-3 (Cell Signalling number 9661S); MAP3K4 (Sigma m7194). Wholemount immunohistochemistry was performed as previously described [106]. Section immunohistochemistry was performed on the basis of protocols described in [39]. Wholemount samples were imaged using a Leica TCS SP5 confocal microscope. Sections were visualised using a Zeiss Axiophot 2.

### Gonadal Cell Proliferation

After immunostaining with anti-PECAM and anti–pHH3 (Upstate, number 06-570) and nuclear counterstaining with aqueous DAPI, the central third of each gonad was imaged using a Leica TCS SP5 confocal microscope (40×). A Z-stack series (10 µm steps) was generated for each sample and then three central sections were chosen for cell counts in the coelomic region (pHH3-positive cells and DAPI-stained nuclei). Sections were separated by 20 µm to ensure that no cell was counted twice. Differences between samples were assessed using a two-tailed *t*-test.

### Organ Culture

Culturing of embryonic gonads and recombination experiments between subdissected gonads and marked mesonephroi were performed based on methodologies described in [50] and [106]. Briefly, XY urogenital ridges (UGRs), consisting of gonad and attached mesonephros, were collected at 11.5 dpc (16–19 ts stage) and cultured to establish conditions under which testis cords formed reliably after 48 h culture. Samples were incubated on 1.5% agar blocks at 37°C/5% CO_2_ in Dulbecco's Minimal Eagle's Medium (DMEM)/10% fetal calf serum (FCS)/50 µg/ml ampicillin/200 mM L-glutamine in the presence of MAPK inhibitors or vehicle control. For recombination cultures, 11.5 dpc XY male UGRs from *byg/byg* mutant embryos were subdissected into component gonad and mesonephros in PBS. The gonads were recombined with mesonephroi from XY Tg(GFPU)5Nagy/J embryos (ubiquitously expressing GFP) and cultured for 48 h, as above. Migration from the marked mesonephros into the attached gonad was imaged using a Leica TCS SP5 confocal microscope. No migration was observed into control XX gonads during these experiments.

The following MAPK signalling inhibitors were used: SB202190 (p38 inhibitor, Sigma) and U0126 (ERK [Mek1] inhibitor, Sigma). SB202190 was used at a final concentration of 25 µM in culture medium, in line with previously reported in vitro studies employing this inhibitor [115]–[117]. U0126 was also used at a final concentration of 25 µM [86],[118],[119].

To examine the effects of exogenous FGF9 expression in XX gonad development we employed the methodology described in [56]. Briefly, agarose beads (Sigma-Aldrich) were incubated in culture medium containing 50 ng/ml FGF9 protein (R&D Systems), or 0.1% BSA, in a humidified chamber at room temperature for at least 5 h. Beads were then placed adjacent to gonads (*n* = 3 for each genotypic class) and cultured for approximately 42 h.

### Ethics Statement

Animal procedures employed in this study were authorized by UK Home Office Project License PPL 30/2381.

## Supporting Information

Figure S1 **Gonadal sex reversal and failure of mesonephric cell migration into XY** ***byg/byg*** **embryonic gonads during organ culture.** (A) In vitro culture of wild-type XY embryonic gonads at 11.5 dpc for 48 h results in testis cord formation visualised by in situ hybridisation with the Sertoli cell marker, *Sox9*. The asterisk indicates *Sox9* expression in the metanephric kidney, which was still attached to this explant when dissected prior to culture. (B) Culture of XY *byg/byg* gonads results in no testis cord formation and very low levels of *Sox9* transcription, which is limited to the gonadal region adjacent to the mesonephros, as in wild-type female gonads. (C) Wild-type explants do not express *Wnt4* in the developing gonad after culture, but do express this marker in the mesonephros. (D) XY *byg/byg* explants exhibit high levels of *Wnt4* expression in the gonad, similar to XX gonads at 13.5 dpc, indicating gonadal sex reversal in cultured XY mutant gonads. (E) Culture of a wild-type XY gonad adjacent to a stage-matched mesonephros derived from a line expressing GFP (recombination) reveals migration of endothelial cells into the gonad to form cord-like structures and an aggregation of cells in the coelomic region. (F) Culture of an XY *byg/byg* gonad with a marked mesonephros reveals negligible cell migration into the gonad (indicated by the region within the white dotted line).
(2.28 MB TIF)

Figure S2 **Analysis of SF1, FOXL2, and FGFR2 protein expression in XY control and** ***byg/byg*** **gonads at 11.5 dpc.** (A, B) Transverse section showing SF1 expression (green) in a large number of somatic cells of control XY gonads (A) and XY *byg/byg* gonads (B). SF1 signal is nuclear in contrast to the cytoplasmic staining of germ cells with PECAM (red). (C–E) FOXL2 is not detected in control XY gonads at this stage (C) but nuclear signal (green) is detected in somatic cells of XY *byg/byg* (D) and control XX gonads (E). (F–J) FGFR2 (green) is expressed in somatic cell nuclei of control XY gonads (F, G). White arrowhead indicates individual nucleus on section counterstained with DAPI (blue). FGFR2 is still detected in XY *byg/byg* gonads (H, I), but signal is restricted to the cytoplasm of somatic cells (arrowhead, I). This cytoplasmic localisation is reminiscent of FGFR2 expression in control XX gonads of the same stage (J). All gonads were from embryos on the C57BL/6J background.
(2.60 MB TIF)

Figure S3 **Immunohistochemical analysis of pMMK4, pMKK7, pp38, and pJNK on transverse sections of wild-type and** ***byg/byg*** **XY gonads at 11.5 dpc.** In each case, the activated MAPK signalling molecule is detected in somatic cells (red), whilst germ cells and endothelial cells are detected by PECAM staining (green). Counterstaining is with DAPI (blue). The gonad is to the left of the dotted line in each image and the mesonephros is to the right.
(2.91 MB TIF)

## Abbreviations

BSA: bovine serum albumin
dpc: days post coitum
ENU: *N*-ethyl-*N*-nitrosourea
ERK: extracellular signal-related kinase
FGF: fibroblast growth factor
MAPK: mitogen-activated protein kinase
MAP3K4: mitogen-activated protein kinase kinase kinase 4
PECAM: platelet/endothelial cell adhesion molecule
qRT-PCR: quantitative real time-PCR
SOX: SRY-like HMG box
SRY: sex-determining region of the Y
*Tas*: T-associated sex reversal
ts: tail somite
WMISH: wholemount in situ hybridisation

## References

[1] Brennan J, Capel B (2004). One tissue, two fates: molecular genetic events that underlie testis versus ovary development.. Nat Rev Genet.

[2] Gubbay J, Collignon J, Koopman P, Capel B, Economou A (1990). A gene mapping to the sex-determining region of the mouse Y chromosome is a member of a novel family of embryonically expressed genes.. Nature.

[3] Koopman P, Münsterberg A, Capel B, Vivian N, Lovell-Badge R (1990). Expression of a candidate sex-determining gene during mouse testis differentiation.. Nature.

[4] Koopman P, Gubbay J, Vivian N, Goodfellow P, Lovell-Badge R (1991). Male development of chromosomally female mice transgenic for *Sry*.. Nature.

[5] Lovell-Badge R, Robertson E (1990). XY female mice resulting from a heritable mutation in the murine primary testis determining gene, *Tdy*.. Development.

[6] Hawkins J. R, Taylor A, Berta P, Levilliers J, van der Auwera B (1992). Mutational analysis of SRY: nonsense and missense mutations in XY sex reversal.. Human Genetics.

[7] Chaboissier M. C, Kobayashi A, Vidal V. I, Lutzkendorf S, van de Kant H. J (2004). Functional analysis of Sox8 and Sox9 during sex determination in the mouse.. Development.

[8] Barrionuevo F, Bagheri-Fam S, Klattig J, Kist R, Taketo M. M (2006). Homozygous inactivation of Sox9 causes complete XY sex reversal in mice.. Biol Reprod.

[9] Meeks J. J, Weiss J, Jameson J. L (2003). Dax1 is required for testis determination.. Nat Genet.

[10] Colvin J. S, Green R. P, Schmahl J, Capel B, Ornitz D. M (2001). Male-to-female sex reversal in mice lacking fibroblast growth factor 9.. Cell.

[11] Kim Y, Bingham N, Sekido R, Parker K. L, Lovell-Badge R (2007). Fibroblast growth factor receptor 2 regulates proliferation and Sertoli differentiation during male sex determination.. Proc Natl Acad Sci U S A.

[12] Bagheri-Fam S, Sim H, Bernard P, Jayakody I, Taketo M. M (2008). Loss of Fgfr2 leads to partial XY sex reversal.. Dev Biol.

[13] Tevosian S. G, Albrecht K. H, Crispino J. D, Fujiwara Y, Eicher E. M (2002). Gonadal differentiation, sex determination and normal Sry expression in mice require direct interaction between transcription partners GATA4 and FOG2.. Development.

[14] Bouma G. J, Washburn L. L, Albrecht K. H, Eicher E. M (2007). Correct dosage of Fog2 and Gata4 transcription factors is critical for fetal testis development in mice.. Proc Natl Acad Sci U S A.

[15] Katoh-Fukui Y, Tsuchiya R, Shiroishi T, Nakahara Y, Hashimoto N (1998). Male-to-female sex reversal in M33 mutant mice.. Nature.

[16] Hammes A, Guo J. K, Lutsch G, Leheste J. R, Landrock D (2001). Two splice variants of the Wilms' tumor 1 gene have distinct functions during sex determination and nephron formation.. Cell.

[17] Nef S, Verma-Kurvari S, Merenmies J, Vassalli J. D, Efstratiadis A (2003). Testis determination requires insulin receptor family function in mice.. Nature.

[18] Bitgood M. J, Shen L, McMahon A. P (1996). Sertoli cell signaling by *Desert Hedgehog* regulates the male germline.. Current Biol.

[19] Clark A. M, Garland K. K, Russell L. D (2000). Desert hedgehog (Dhh) gene is required in the mouse testis for formation of adult-type Leydig cells and normal development of peritubular cells and seminiferous tubules.. Biol Reprod.

[20] Yao H. H, Whoriskey W, Capel B (2002). Desert Hedgehog/Patched 1 signaling specifies fetal Leydig cell fate in testis organogenesis.. Genes Dev.

[21] Brennan J, Tilmann C, Capel B (2003). Pdgfr-alpha mediates testis cord organization and fetal Leydig cell development in the XY gonad.. Genes Dev.

[22] Cui S, Ross A, Stallings N, Parker K. L, Capel B (2004). Disrupted gonadogenesis and male-to-female sex reversal in Pod1 knockout mice.. Development.

[23] Kitamura K, Yanazawa M, Sugiyama N, Miura H, Iizuka-Kogo A (2002). Mutation of ARX causes abnormal development of forebrain and testes in mice and X-linked lissencephaly with abnormal genitalia in humans.. Nat Genet.

[24] Jeays-Ward K, Dandonneau M, Swain A (2004). Wnt4 is required for proper male as well as female sexual development.. Dev Biol.

[25] Chi L, Itaranta P, Zhang S, Vainio S (2006). Sprouty2 is involved in male sex organogenesis by controlling fibroblast growth factor 9-induced mesonephric cell migration to the developing testis.. Endocrinology.

[26] Park S. Y, Meeks J. J, Raverot G, Pfaff L. E, Weiss J (2005). Nuclear receptors Sf1 and Dax1 function cooperatively to mediate somatic cell differentiation during testis development.. Development.

[27] Raymond C. S, Murphy M. W, O'Sullivan M. G, Bardwell V. J, Zarkower D (2000). Dmrt1, a gene related to worm and fly sexual regulators, is required for mammalian testis differentiation.. Genes Dev.

[28] Eicher E. M, Washburn L. L, Schork N. J, Lee B. K, Shown E. P (1996). Sex-determining genes on mouse autosomes identified by linkage analysis of C57BL/6-YPos sex reversal.. Nature Genetics.

[29] Bouma G. J, Albrecht K. H, Washburn L. L, Recknagel A. K, Churchill G. A (2005). Gonadal sex reversal in mutant Dax1 XY mice: a failure to upregulate Sox9 in pre-Sertoli cells.. Development.

[30] Qin Y, Poirier C, Truong C, Schumacher A, Agoulnik A. I (2003). A major locus on mouse chromosome 18 controls XX sex reversal in Odd Sex (Ods) mice.. Hum Mol Genet.

[31] Grimmond S, Van Hateren N, Siggers P, Arkell R, Larder R (2000). Sexually dimorphic expression of protease nexin-1 and vanin-1 in the developing mouse gonad prior to overt differentiation suggests a role in mammalian sexual development.. Hum Mol Genet.

[32] Bowles J, Bullejos M, Koopman P (2000). A subtractive gene expression screen suggests a role for vanin-1 in testis development in mice.. Genesis.

[33] Nef S, Schaad O, Stallings N. R, Cederroth C. R, Pitetti J. L (2005). Gene expression during sex determination reveals a robust female genetic program at the onset of ovarian development.. Dev Biol.

[34] Beverdam A, Koopman P (2006). Expression profiling of purified mouse gonadal somatic cells during the critical time window of sex determination reveals novel candidate genes for human sexual dysgenesis syndromes.. Hum Mol Genet.

[35] Bouma G. J, Affourtit J. P, Bult C. J, Eicher E. M (2006). Transcriptional profile of mouse pre-granulosa and Sertoli cells isolated from early-differentiated fetal gonads.. Gene Expr Patterns.

[36] Bowles J, Knight D, Smith C, Wilhelm D, Richman J (2006). Retinoid signaling determines germ cell fate in mice.. Science.

[37] Koubova J, Menke D. B, Zhou Q, Capel B, Griswold M. D (2006). Retinoic acid regulates sex-specific timing of meiotic initiation in mice.. Proc Natl Acad Sci U S A.

[38] Adams I. R, McLaren A (2002). Sexually dimorphic development of mouse primordial germ cells: switching from oogenesis to spermatogenesis.. Development.

[39] Wilhelm D, Martinson F, Bradford S, Wilson M. J, Combes A. N (2005). Sertoli cell differentiation is induced both cell-autonomously and through prostaglandin signaling during mammalian sex determination.. Dev Biol.

[40] Washburn L. L, Eicher E. M (1983). Sex reversal in XY mice caused by dominant mutation on chromosome 17.. Nature.

[41] Washburn L. L, Lee B. K, Eicher E. M (1990). Inheritance of T-associated sex reversal in mice.. Genet Res.

[42] Gerwins P, Blank J. L, Johnson G. L (1997). Cloning of a novel mitogen-activated protein kinase kinase kinase, MEKK4, that selectively regulates the c-Jun amino terminal kinase pathway.. J Biol Chem.

[43] Takekawa M, Posas F, Saito H (1997). A human homolog of the yeast Ssk2/Ssk22 MAP kinase kinase kinases, MTK1, mediates stress-induced activation of the p38 and JNK pathways.. EMBO J.

[44] Chi H, Sarkisian M. R, Rakic P, Flavell R. A (2005). Loss of mitogen-activated protein kinase kinase kinase 4 (MEKK4) results in enhanced apoptosis and defective neural tube development.. Proc Natl Acad Sci U S A.

[45] Abell A. N, Rivera-Perez J. A, Cuevas B. D, Uhlik M. T, Sather S (2005). Ablation of MEKK4 kinase activity causes neurulation and skeletal patterning defects in the mouse embryo.. Mol Cell Biol.

[46] Brennan J, Karl J, Martineau J, Nordqvist K, Schmahl J (1998). Sry and the testis: molecular pathways of organogenesis.. J Exp Zool.

[47] Capel B (2000). The battle of the sexes.. Mech Dev.

[48] Schmahl J, Eicher E. M, Washburn L. L, Capel B (2000). Sry induces cell proliferation in the mouse gonad.. Development.

[49] Schmahl J, Capel B (2003). Cell proliferation is necessary for the determination of male fate in the gonad.. Dev Biol.

[50] Martineau J, Nordqvist K, Tilmann C, Lovell-Badge R, Capel B (1997). Male-specific cell migration into the developing gonad.. Curr Biol.

[51] Capel B, Albrecht K. H, Washburn L. L, Eicher E. M (1999). Migration of mesonephric cells into the mammalian gonad depends on Sry.. Mech Dev.

[52] Tilmann C, Capel B (1999). Mesonephric cell migration induces testis cord formation and sertoli cell differentiation in the mammalian gonad.. Development.

[53] Albrecht K. H, Capel B, Washburn L. L, Eicher E. M (2000). Defective mesonephric cell migration is associated with abnormal testis cord development in C57BL/6J XY(Mus domesticus) mice.. Dev Biol.

[54] Sekido R, Bar I, Narvaez V, Penny G, Lovell-Badge R (2004). SOX9 is up-regulated by the transient expression of SRY specifically in Sertoli cell precursors.. Dev Biol.

[55] Sekido R, Lovell-Badge R (2008). Sex determination involves synergistic action of SRY and SF1 on a specific Sox9 enhancer.. Nature.

[56] Kim Y, Kobayashi A, Sekido R, DiNapoli L, Brennan J (2006). Fgf9 and Wnt4 act as antagonistic signals to regulate mammalian sex determination.. PLoS Biol.

[57] Malki S, Nef S, Notarnicola C, Thevenet L, Gasca S (2005). Prostaglandin D2 induces nuclear import of the sex-determining factor SOX9 via its cAMP-PKA phosphorylation.. Embo J.

[58] Moniot B, Declosmenil F, Barrionuevo F, Scherer G, Aritake K (2009). The PGD2 pathway, independently of FGF9, amplifies SOX9 activity in Sertoli cells during male sexual differentiation.. Development.

[59] Arango N. A, Lovell-Badge R, Behringer R. R (1999). Targeted mutagenesis of the endogenous mouse Mis gene promoter: in vivo definition of genetic pathways of vertebrate sexual development.. Cell.

[60] Wilson M. J, Jeyasuria P, Parker K, Koopman P (2004). The transcription factors steroidogenic factor-1 and SOX9 regulate expression of Vanin-1 during mouse testis development.. J Biol Chem.

[61] Schmahl J, Kim Y, Colvin J. S, Ornitz D. M, Capel B (2004). Fgf9 induces proliferation and nuclear localization of FGFR2 in Sertoli precursors during male sex determination.. Development.

[62] Schmidt D, Ovitt C. E, Anlag K, Fehsenfeld S, Gredsted L (2004). The murine winged-helix transcription factor Foxl2 is required for granulosa cell differentiation and ovary maintenance.. Development.

[63] Uda M, Ottolenghi C, Crisponi L, Garcia J. E, Deiana M (2004). Foxl2 disruption causes mouse ovarian failure by pervasive blockage of follicle development.. Hum Mol Genet.

[64] Ottolenghi C, Omari S, Garcia-Ortiz J. E, Uda M, Crisponi L (2005). Foxl2 is required for commitment to ovary differentiation.. Hum Mol Genet.

[65] Ottolenghi C, Pelosi E, Tran J, Colombino M, Douglass E (2007). Loss of Wnt4 and Foxl2 leads to female-to-male sex reversal extending to germ cells.. Hum Mol Genet.

[66] Loffler K. A, Zarkower D, Koopman P (2003). Etiology of ovarian failure in blepharophimosis ptosis epicanthus inversus syndrome: FOXL2 is a conserved, early-acting gene in vertebrate ovarian development.. Endocrinology.

[67] Gao F, Maiti S, Alam N, Zhang Z, Deng J. M (2006). The Wilms tumor gene, Wt1, is required for Sox9 expression and maintenance of tubular architecture in the developing testis.. Proc Natl Acad Sci U S A.

[68] Bradford S. T, Wilhelm D, Koopman P (2007). Comparative analysis of anti-mouse SRY antibodies.. Sex Dev.

[69] Bullejos M, Koopman P (2005). Delayed Sry and Sox9 expression in developing mouse gonads underlies B6-Y(DOM) sex reversal.. Dev Biol.

[70] Hiramatsu R, Matoba S, Kanai-Azuma M, Tsunekawa N, Katoh-Fukui Y (2008). A critical time window of Sry action in gonadal sex determination in mice.. Development.

[71] Cuevas B. D, Abell A. N, Johnson G. L (2007). Role of mitogen-activated protein kinase kinase kinases in signal integration.. Oncogene.

[72] Winter-Vann A. M, Johnson G. L (2007). Integrated activation of MAP3Ks balances cell fate in response to stress.. J Cell Biochem.

[73] Davies S. P, Reddy H, Caivano M, Cohen P (2000). Specificity and mechanism of action of some commonly used protein kinase inhibitors.. Biochem J.

[74] Uzumcu M, Westfall S. D, Dirks K. A, Skinner M. K (2002). Embryonic testis cord formation and mesonephric cell migration requires the phosphotidylinositol 3-kinase signaling pathway.. Biol Reprod.

[75] Gazel A, Nijhawan R. I, Walsh R, Blumenberg M (2008). Transcriptional profiling defines the roles of ERK and p38 kinases in epidermal keratinocytes.. J Cell Physiol.

[76] Mann E. A, Silver L. M, Elliott R. W (1986). Genetic analysis of a mouse t complex locus that is homologous to a kidney cDNA clone.. Genetics.

[77] Albrecht K. H, Young M, Washburn L. L, Eicher E. M (2003). Sry expression level and protein isoform differences play a role in abnormal testis development in C57BL/6J mice carrying certain Sry alleles.. Genetics.

[78] Karl J, Capel B (1998). Sertoli cells of the mouse testis originate from the coelomic epithelium.. Dev Biol.

[79] Sekido R, Lovell-Badge R (2009). Sex determination and SRY: down to a wink and a nudge?. Trends Genet.

[80] Hammer G. D, Krylova I, Zhang Y, Darimont B. D, Simpson K (1999). Phosphorylation of the nuclear receptor SF-1 modulates cofactor recruitment: integration of hormone signaling in reproduction and stress.. Mol Cell.

[81] Desclozeaux M, Krylova I. N, Horn F, Fletterick R. J, Ingraham H. A (2002). Phosphorylation and intramolecular stabilization of the ligand binding domain in the nuclear receptor steroidogenic factor 1.. Mol Cell Biol.

[82] Charron F, Tsimiklis G, Arcand M, Robitaille L, Liang Q (2001). Tissue-specific GATA factors are transcriptional effectors of the small GTPase RhoA.. Genes Dev.

[83] Desclozeaux M, Poulat F, de Santa Barbara P, Capony J. P, Turowski P (1998). Phosphorylation of an N-terminal motif enhances DNA-binding activity of the human SRY protein.. J Biol Chem.

[84] Matsuyama M, Mizusaki H, Shimono A, Mukai T, Okumura K (2005). A novel isoform of Vinexin, Vinexin gamma, regulates Sox9 gene expression through activation of MAPK cascade in mouse fetal gonad.. Genes Cells.

[85] Hotokezaka H, Sakai E, Kanaoka K, Saito K, Matsuo K (2002). U0126 and PD98059, specific inhibitors of MEK, accelerate differentiation of RAW264.7 cells into osteoclast-like cells.. J Biol Chem.

[86] Murakami S, Kan M, McKeehan W. L, de Crombrugghe B (2000). Up-regulation of the chondrogenic Sox9 gene by fibroblast growth factors is mediated by the mitogen-activated protein kinase pathway.. Proc Natl Acad Sci U S A.

[87] Tew S. R, Hardingham T. E (2006). Regulation of SOX9 mRNA in human articular chondrocytes involving p38 MAPK activation and mRNA stabilization.. J Biol Chem.

[88] Adams R. H, Porras A, Alonso G, Jones M, Vintersten K (2000). Essential role of p38alpha MAP kinase in placental but not embryonic cardiovascular development.. Mol Cell.

[89] Schlessinger J (2000). Cell signaling by receptor tyrosine kinases.. Cell.

[90] Humpert P. M, Djuric Z, Zeuge U, Oikonomou D, Seregin Y (2008). Insulin stimulates the clonogenic potential of angiogenic endothelial progenitor cells by IGF-1 receptor-dependent signaling.. Mol Med.

[91] Zhang X, Lin M, van Golen K. L, Yoshioka K, Itoh K (2005). Multiple signaling pathways are activated during insulin-like growth factor-I (IGF-I) stimulated breast cancer cell migration.. Breast Cancer Res Treat.

[92] Kayali A. G, Stotland A, Gunst K. V, Kritzik M, Liu G (2005). Growth factor-induced signaling of the pancreatic epithelium.. J Endocrinol.

[93] Pettipher R, Hansel T. T, Armer R (2007). Antagonism of the prostaglandin D2 receptors DP1 and CRTH2 as an approach to treat allergic diseases.. Nat Rev Drug Discov.

[94] Fernandez-Tome M, Favale N, Kraemer L, Gabriela Marquez M, Speziale E (2004). p44/42(ERK1/2) MAPK and PLD activation by PGD2 preserves papillary phosphatidylcholine homeostasis.. Biochem Biophys Res Commun.

[95] Chiba T, Kanda A, Ueki S, Ito W, Kamada Y (2006). Prostaglandin D2 induces IL-8 and GM-CSF by bronchial epithelial cells in a CRTH2-independent pathway.. Int Arch Allergy Immunol.

[96] Dan I, Watanabe N. M, Kusumi A (2001). The Ste20 group kinases as regulators of MAP kinase cascades.. Trends Cell Biol.

[97] Cattanach B. M (1987). Sex-reversed mice and sex determination.. Ann N Y Acad Sci.

[98] Hastie N. D (1992). Dominant negative mutations in the Wilms tumour (WT1) gene cause Denys-Drash syndrome–proof that a tumour-suppressor gene plays a crucial role in normal genitourinary development.. Hum Mol Genet.

[99] Achermann J. C, Ito M, Hindmarsh P. C, Jameson J. L (1999). A mutation in the gene encoding steroidogenic factor-1 causes XY sex reversal and adrenal failure in humans [letter].. Nat Genet.

[100] Ostrer H (2001). Identifying genes for male sex determination in humans.. J Exp Zool.

[101] Washburn L. L, Albrecht K. H, Eicher E. M (2001). C57BL/6J-T-associated sex reversal in mice is caused by reduced expression of a Mus domesticus Sry allele.. Genetics.

[102] Marsh L, Neiman A. M, Herskowitz I (1991). Signal transduction during pheromone response in yeast.. Annu Rev Cell Biol.

[103] Feldbrugge M, Kamper J, Steinberg G, Kahmann R (2004). Regulation of mating and pathogenic development in Ustilago maydis.. Curr Opin Microbiol.

[104] Casselton L. A (2008). Fungal sex genes-searching for the ancestors.. Bioessays.

[105] Patterson V. L, Damrau C, Grimes D. T, Paudyal A, Reeve B (2009). Mouse hitchhiker mutants have spina bifida, dorso-ventral patterning defects and polydactyly: Identification of Tulp3 as a novel negative regulator of the Sonic hedghog pathway.. Hum Mol Genet.

[106] Warr N, Siggers P, Bogani D, Brixey R, Pastorelli L (2009). Sfrp1 and Sfrp2 are required for normal male sexual development in mice.. Dev Biol.

[107] Chi H, Lu B, Takekawa M, Davis R. J, Flavell R. A (2004). GADD45beta/GADD45gamma and MEKK4 comprise a genetic pathway mediating STAT4-independent IFNgamma production in T cells.. EMBO J.

[108] Johnson D. R (1974). Hairpin-tail: a case of post-reductional gene action in the mouse egg.. Genetics.

[109] Prager E. M, Boursot P, Sage R. D (1997). New assays for Y chromosome and p53 pseudogene clines among East Holstein house mice.. Mamm Genome.

[110] Wright E, Hargrave M. R, Christiansen J, Cooper L, Kun J (1995). The *Sry*-related gene *Sox-9* is expressed during chondrogenesis in mouse embryos.. Nature Genetics.

[111] Siggers P, Smith L, Greenfield A (2002). Sexually dimorphic expression of Gata-2 during mouse gonad development.. Mech Dev.

[112] Smith L, Willan J, Warr N, Brook F. A, Cheeseman M (2008). The Maestro (Mro) gene is dispensable for normal sexual development and fertility in mice.. PLoS ONE.

[113] Bullejos M, Koopman P (2001). Spatially dynamic expression of Sry in mouse genital ridges.. Dev Dyn.

[114] Cocquet J, Pailhoux E, Jaubert F, Servel N, Xia X (2002). Evolution and expression of FOXL2.. J Med Genet.

[115] Park J. M, Greten F. R, Li Z. W, Karin M (2002). Macrophage apoptosis by anthrax lethal factor through p38 MAP kinase inhibition.. Science.

[116] Chan W. K, Dickerson A, Ortiz D, Pimenta A. F, Moran C. M (2004). Mitogen-activated protein kinase regulates neurofilament axonal transport.. J Cell Sci.

[117] Jacquel A, Herrant M, Defamie V, Belhacene N, Colosetti P (2006). A survey of the signaling pathways involved in megakaryocytic differentiation of the human K562 leukemia cell line by molecular and c-DNA array analysis.. Oncogene.

[118] Shi Y, Sahu R. P, Srivastava S. K (2008). Triphala inhibits both in vitro and in vivo xenograft growth of pancreatic tumor cells by inducing apoptosis.. BMC Cancer.

[119] Thomsen M. K, Butler C. M, Shen M. M, Swain A (2008). Sox9 is required for prostate development.. Dev Biol.

