# Supplementary figures and images for: Loss of Mitogen-Activated Protein Kinase Kinase Kinase 4 (MAP3K4) Reveals a Requirement for MAPK Signalling in Mouse Sex Determination
Source: PLoS Biol. 2009 Sep 15;7(9):e1000196. doi: 10.1371/journal.pbio.1000196 (PMC2733150; doi:10.1371/journal.pbio.1000196)

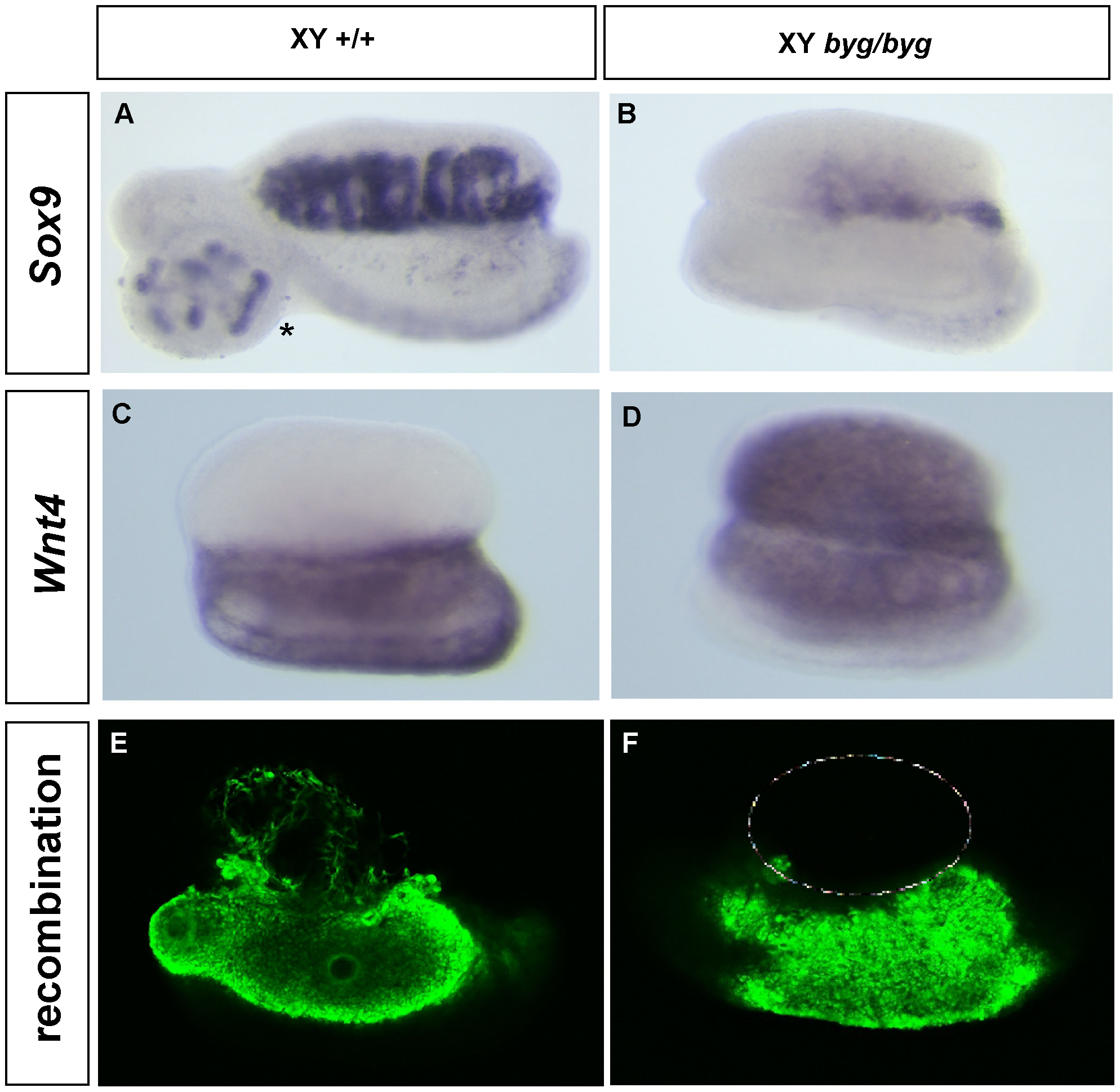

Supplement: Figure S1 — Gonadal sex reversal and failure of mesonephric cell migration into XY byg/byg embryonic gonads during organ culture. (A) In vitro culture of wild-type XY embryonic gonads at 11.5 dpc for 48 h results in testis cord formation visualised by in situ hybridisation with the Sertoli cell marker, Sox9. The asterisk indicates Sox9 expression in the metanephric kidney, which was still attached to this explant when dissected prior to culture. (B) Culture of XY byg/byg gonads results in no testis cord formation and very low levels of Sox9 transcription, which is limited to the gonadal region adjacent to the mesonephros, as in wild-type female gonads. (C) Wild-type explants do not express Wnt4 in the developing gonad after culture, but do express this marker in the mesonephros. (D) XY byg/byg explants exhibit high levels of Wnt4 expression in the gonad, similar to XX gonads at 13.5 dpc, indicating gonadal sex reversal in cultured XY mutant gonads. (E) Culture of a wild-type XY gonad adjacent to a stage-matched mesonephros derived from a line expressing GFP (recombination) reveals migration of endothelial cells into the gonad to form cord-like structures and an aggregation of cells in the coelomic region. (F) Culture of an XY byg/byg gonad with a marked mesonephros reveals negligible cell migration into the gonad (indicated by the region within the white dotted line). (2.28 MB TIF) [file pbio.1000196.s001.tif]

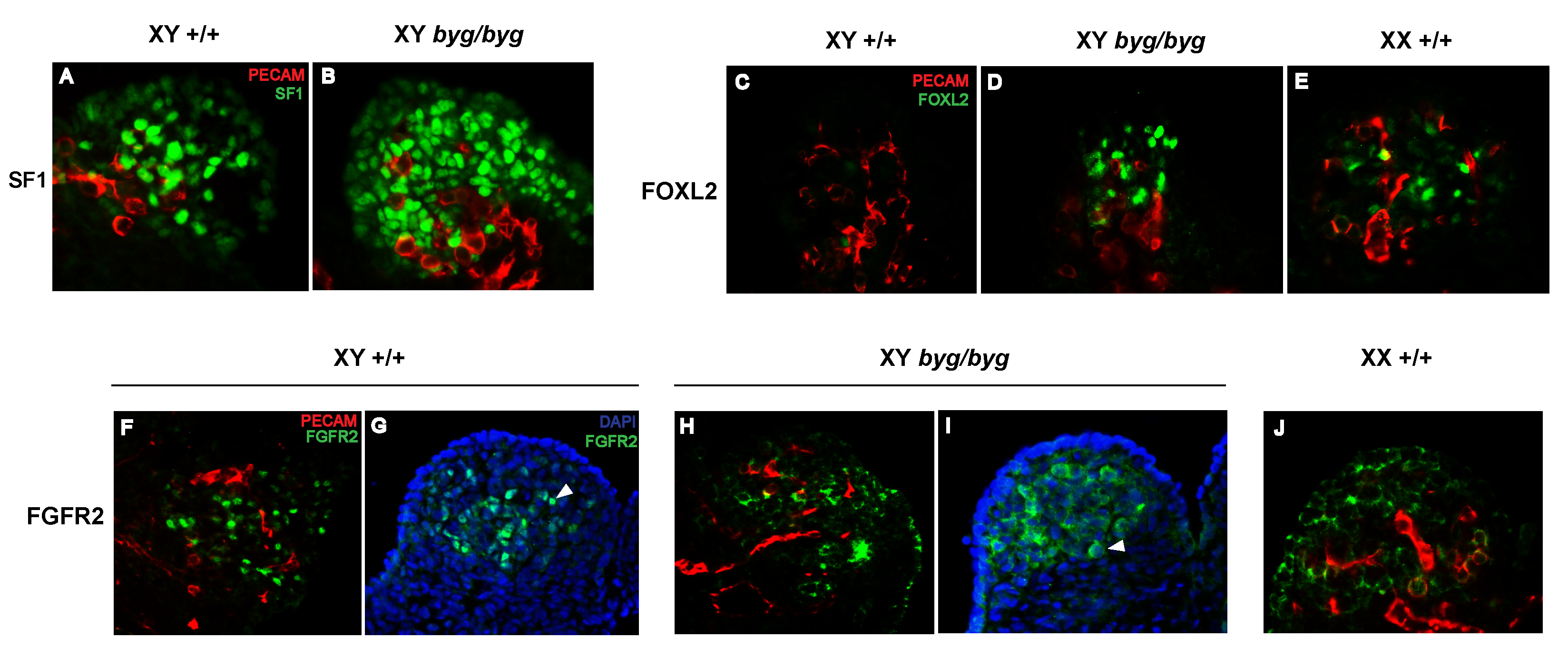

Supplement: Figure S2 — Analysis of SF1, FOXL2, and FGFR2 protein expression in XY control and byg/byg gonads at 11.5 dpc. (A, B) Transverse section showing SF1 expression (green) in a large number of somatic cells of control XY gonads (A) and XY byg/byg gonads (B). SF1 signal is nuclear in contrast to the cytoplasmic staining of germ cells with PECAM (red). (C–E) FOXL2 is not detected in control XY gonads at this stage (C) but nuclear signal (green) is detected in somatic cells of XY byg/byg (D) and control XX gonads (E). (F–J) FGFR2 (green) is expressed in somatic cell nuclei of control XY gonads (F, G). White arrowhead indicates individual nucleus on section counterstained with DAPI (blue). FGFR2 is still detected in XY byg/byg gonads (H, I), but signal is restricted to the cytoplasm of somatic cells (arrowhead, I). This cytoplasmic localisation is reminiscent of FGFR2 expression in control XX gonads of the same stage (J). All gonads were from embryos on the C57BL/6J background. (2.60 MB TIF) [file pbio.1000196.s002.tif]

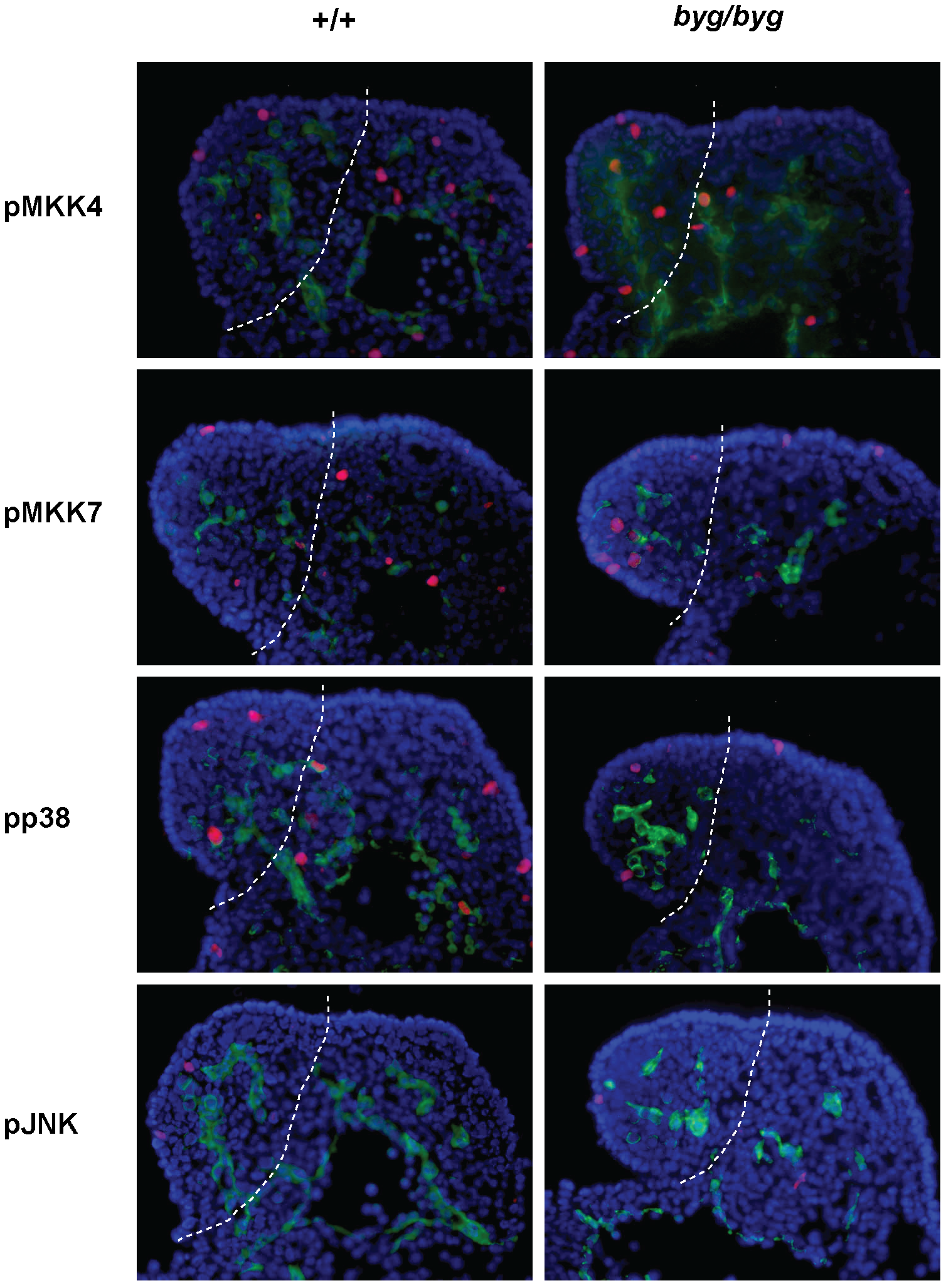

Supplement: Figure S3 — Immunohistochemical analysis of pMMK4, pMKK7, pp38, and pJNK on transverse sections of wild-type and byg/byg XY gonads at 11.5 dpc. In each case, the activated MAPK signalling molecule is detected in somatic cells (red), whilst germ cells and endothelial cells are detected by PECAM staining (green). Counterstaining is with DAPI (blue). The gonad is to the left of the dotted line in each image and the mesonephros is to the right. (2.91 MB TIF) [file pbio.1000196.s003.tif]
